# Targeting Bone Cells During Sexual Maturation Reveals Sexually Dimorphic Regulation of Endochondral Ossification

**DOI:** 10.1002/jbm4.10413

**Published:** 2020-10-14

**Authors:** Heather Fairfield, Samantha Costa, Victoria DeMambro, Celine Schott, Janaina Da Silva Martins, Mathieu Ferron, Calvin Vary, Michaela R Reagan

**Affiliations:** ^1^ Center for Molecular Medicine, Maine Medical Center Research Institute Scarborough ME USA; ^2^ University of Maine Graduate School of Biomedical Science and Engineering Orono ME USA; ^3^ Graduate School of Biomedical Sciences and School of Medicine Tufts University Boston MA USA; ^4^ Molecular Physiology Research Unit Institut de Recherches Cliniques de Montreal Montreal Quebec Canada; ^5^ Department of Medicine and Molecular Biology Programs of the Faculty of Medicine Université de Montreal Montreal Quebec Canada

**Keywords:** BONE–FAT INTERACTIONS, CHONDROCYTES, CRE, DIPHTHERIA TOXIN, GENETIC ANIMAL MODELS, GROWTH PLATE, OSTEOCALCIN, OSTEOCYTES

## Abstract

In endochondral ossification, chondroblasts become embedded in their matrix and become chondrocytes, which are mature cells that continue to proliferate, eventually becoming hypertrophic. Hypertrophic chondrocytes produce cartilage that is then resorbed by osteoclasts prior to bone matrix replacement via osteoblasts. Although sexually dimorphic bone phenotypes have long been characterized, specific modulation of the growth plate during a critical window in sexual maturation has not been evaluated. Here we report that specific depletion of osteocalcin‐ (OCN‐) expressing cells in vivo during sexual maturation leads to dimorphic bone phenotypes in males and females. At 6 to 8 weeks of age, OCN‐Cre;iDTR (inducible diphtheria toxin receptor‐expressing) mice were treated with diphtheria toxin (DT) for 2 weeks to deplete OCN+ cells. At the end of the study, long bones were collected for μCT and histomorphometry, and serum was collected for proteomic and lipidomic analyses. Ablation of OCN+ cells in mice leads to consistent trends for weight loss after 2 weeks of treatment. Females exhibited decreased skeletal parameters in response to OCN+ cell ablation treatment, as expected. However, OCN+ cell ablation in males uniquely displayed an expansion of hypertrophic chondrocytes, a widening of the growth plate, and an abnormal “clubbing” anatomy of the distal femur. Following DT treatment, mice from both sexes also underwent metabolic cage analysis, in which both sexes exhibited decreased energy expenditure. We conclude that skewing endochondral bone formation during longitudinal growth has a profound effect on body weight and energy expenditure with sex‐specific effects on developing bone. © 2020 The Authors. *JBMR Plus* published by Wiley Periodicals LLC on behalf of American Society for Bone and Mineral Research.

## Introduction

Sexual differences in the longitudinal growth of the skeleton during childhood and puberty have long been acknowledged in mammals.^(^ ^1^ ^)^ In humans, the pubertal growth spurt, growth plate closure, and bone mass accrual in males are primarily under the control of estrogens; however, treatment with dihydrotestosterone (a nonaromatized androgen) also resulted in increased ulnar bone length in prepubertal children.^(^ ^1^ ^)^ In mice, dynamic histomorphometry has revealed differences in bone formation rates: with a higher bone formation rate in males than in females.^(^ ^2^ ^)^ Traditionally, androgens (stimulatory) and estrogens (inhibitory) were thought to regulate these dimorphisms; however, testosterone in pubertal males can be aromatized into 17β‐estradiol and can therefore signal via the androgen receptor or estrogen receptor (ER) α or β.^(^ ^1^ ^)^ Multiple studies in mice suggest that these processes are limited temporally with estrogens acting during early puberty in females, and androgens acting during late puberty in males.^(^ ^1^ ^)^ Additionally, androgens are critical regulators of trabecular bone in males regardless of the presence (or absence) of either aromatase inhibitors or ERα; however, functional studies in females underlie the importance of estrogen‐ERα signaling in their bone formation.^(^ ^1^, ^3^ ^)^

Bone develops through two main processes: intramembranous ossification and endochondral ossification. The first process involves differentiation of mesenchymal stem cells (MSCs) into osteoblasts to generate new “woven” bone. The second process involves differentiation of MSCs into prechondroblasts and chondroblasts to form cartilaginous matrix.^(^ ^4^ ^)^ In endochondral ossification, chondroblasts become embedded in their matrix and become chondrocytes, which are mature cells that continue to proliferate, eventually becoming hypertrophic. Chondrocyte differentiation is regulated by a number of factors including Indian hedgehog, FGFs, BMPs, and parathyroid hormone‐related peptide: Many of which control MSC commitment through Runx2, Sox9, and the Wnt signaling pathways.^(^ ^4^ ^)^ In addition, multiple studies suggest that estrogen signaling through ERα also modulates endochondral ossification through direct signaling in chondrocytes.^(^ ^1^ ^)^ Studies have shown that the androgen receptor has been detected in mammalian chondrocytes, and that these cells respond to androgen stimulation.^(^ ^5^ ^)^ During endochondral bone formation, chondrocytes produce cartilage that is then resorbed by osteoclasts prior to bone matrix replacement via osteoblasts (OBs). The regulation of osteoclasts is controlled by multiple cell types in the BM niche, by their production of the cytokine RANKL, which stimulates osteoclast differentiation and triggers osteoclast activation, and osteoprotegerin, the RANK decoy. Over the past decade, multiple studies have linked bone cells—and OBs in particular, by way of osteocalcin (OCN) production—to processes such as energy metabolism, reproduction, and beyond.^(^ ^6^, ^7^, ^8^, ^9^ ^)^

To examine the general role of OBs, osteocytes, and hypertrophic chondrocytes during a critical window of longitudinal growth, we used the OCN‐Cre;iDTR (osteocalcin‐driven Cre recombinase; inducible diphtheria toxin receptor) model to deplete these cell types (which specifically express OCN) in males and females. Previous studies have used this model to specifically delete mature OBs and osteocytes, with daily injections of diphtheria toxin (DT) in either young mice^(^ ^10^ ^)^ or with no description of age or sex used.^(^ ^11^ ^)^ In the present study, we observed a potent effect of DT treatment in OCN‐Cre;iDTR mice systemically with differential effects on bone in male mice versus female mice. We propose that skewing endochondral bone formation during longitudinal growth negatively affects animal physiology and health with sex‐specific effects on developing bone.

## Materials and Methods

### Mice

Transgenic mice carrying OCN‐Cre crossed with iDTR‐expressing mice, both on a C57BL/6J background, were donated by Dr Scadden's laboratory (Harvard University, Cambridge, MA, USA) to Dr Ghobrial's laboratory (Dana‐Farber Cancer Institute, Boston), then rederived, bred, and used for experiments at the Maine Medical Center Research Institute (MMCRI; Scarborough, ME, USA). C57BL/6J mice were purchased from The Jackson Laboratory (Bar Harbor, ME, USA). All mice were weaned at 21 days after birth, group‐housed thereafter, and fed standard chow (4% fat) and sterile water. All mice used for this study were OCN‐Cre^+^, DTR^mut/mut^ to eliminate any potential variability based on genotype. Following genotyping, mice were randomly assigned to each group prior to administration of DT (0.0125 mg/kg as has previously been described)^(^ ^10^ ^)^ or vehicle volume equivalent (sterile PBS). In all experiments, injections were delivered every other day via i.p. injections, with a maximum of three injections per week for 2 weeks, with the exception of the metabolic cage experiments where the duration of dosing was only 1 week. Over this time, body weights were measured and recorded, and overall health status and body conditioning score was monitored throughout the duration of DT treatments. AquaPak HydroGel (clear H_2_O; South Portland, ME, USA) and moistened grain were provided on the bottom of all cages throughout the duration of applicable experiments. All experimental studies and procedures involving mice were performed in accordance with protocols approved by the governing institutional animal care and use committee. Additional details can be found in the Supplemental Methods.

### Static and dynamic bone histomorphometry and histology

All static and dynamic histomorphometric analyses were performed on femurs and reported according to the criteria established by the ASBMR^(^ ^12^ ^)^ by the Harvard Center for Skeletal Research Histomorphometry Core as has previously been reported.^(^ ^11^ ^)^ Static parameters were measured in the distal femoral metaphysis 0.2 mm below the epiphyseal growth plate, using an Osteomeasure image analyzer (Osteometrics, Atlanta, GA, USA).

### Microcomputed tomography and osmium tetroxide

A high‐resolution desktop μCT system (μCT40; Scanco Medical AG, Brüttisellen, Switzerland) was used to assess trabecular bone microarchitecture and cortical bone morphology, in the proximal metaphysis and mid‐diaphysis of the tibia (*n* = 10 mice). Scans of the entire tibia were acquired using a 10‐μm^3^ isotropic voxel size, 70‐kVp peak X‐ray tube potential, 114‐μA X‐ray intensity, with 200‐ms integration time, and were subjected to Gaussian filtration and segmentation. Image acquisition and analysis protocols adhered to guidelines for μCT assessment of bone microstructure,^(13^ ^)^ and were executed as previously described.^(^ ^14^ ^)^ Quantification and visualization of bone marrow adipose tissue (BMAT) was performed as we have previously described,^(15^ ^)^ utilizing osmium tetroxide stain and μCT. Additional details can be found in the Supplemental Methods.

### Body composition

Dual‐energy X‐ray absorptiometry for whole‐body composition, excluding the head, was performed on mice immediately following treatments or at the end of the recovery period using PIXImus (GE Lunar, Fairfield, CT, USA) as described previously.^(^ ^14^ ^)^

### Serum lipidomic and proteomic analysis

Proteomic and lipidomic analyses were performed on mouse sera from blood collected retro‐orbitally, similar to what we have previously described.^(^ ^14^, ^16^ ^)^ Lipidomic and proteomic analyses were completed using a 5600 TripleTOF mass spectrometer (Sciex, Framingham, MA, USA). Downstream analyses, including *t* tests and principal component analyses, were completed for both data sets using Sciex MarkerView software, essentially as has been described.^(^ ^17^ ^)^ Lipids were analyzed using a global, bias‐free lipid‐profiling acquisition technique (MS/MS^ALL^) as has previously been described.^(^ ^14^ ^)^ Protein profiling of blood sera was completed with sequential window acquisition of all theoretical spectra using a data‐independent method,^(^ ^16^ ^)^ and was analyzed using a mouse‐specific ion library, which employs multiple fragment ion chromatograms for each protein's tryptic peptides. To identify lipids and proteins that were significantly different between DT‐treated animals without recovery and all others, a cut‐off of *p* ≤ .05 was used; minimal cut‐offs for signal intensity were set at ≥100 for lipid and ≥10,000 for protein. Proteins found significantly upregulated in DT‐treated mice without recovery, when compared with all other groups, were determined via *t* test (*p* < .05). Mouse gene symbols for overexpressed proteins were entered into the PANTHER (Protein ANalysis THrough Evolutionary Relationships) database for pathway analysis.

### Serum‐collection methods for chemistry panel

Male and female mice were euthanized through CO_2_ inhalation. Immediately following, approximately 1 mL of blood was collected through a cardiac puncture, placed at room temperature for 1 hour to induce clotting, and centrifuged at 153rcf for 15 min to separate serum from cells. A minimum of 270 μL of serum was collected per sample. The samples were processed by the University of Michigan, Ann Arbor, as part of the National Mouse Metabolic Phenotyping Center program of the National Institute of Diabetes and Digestive and Kidney Diseases.

### Carboxylated and total mouse OCN ELISA

Carboxylated (Gla) and total mouse OCN were measured in serum collected before and 1 or 2 weeks after administration of DT, using two specific ELISA assays as has been previously described.^(^ ^18^ ^)^ Briefly, 96‐well ELISA plates (R&D Systems, Minneapolis, MN, USA) were coated with 100 μL of anti‐Gla‐OCN (2 μg/mL) or anti‐Mid‐OCN (1.5 μg/mL) diluted in 1X antibody coating buffer (Immunochemistry Technologies, Bloomington, MN, USA) and incubated overnight at room temperature. Then, the plates were washed two times with wash buffer (0.1% Tween in PBS) and blocked with assay diluent (0.1% Tween and 3% BSA in PBS) for 4 hours at room temperature. Afterwards, 2 μL of standard (synthetic Gla‐OCN) or diluted serum samples (1:10 dilution in assay diluent) in 98 μL of assay diluent was added, and the plates sealed and placed overnight at 4°C. The next day, the plates were washed five times and 100 μL of horseradish peroxidase‐conjugated anti‐CT‐OCN (C‐terminal region of osteocalcin) (1 μg/mL in assay diluent) was added to each well and incubated on a shaker (~200–300 rpm) for 1 hour at room temperature. Following six washes, detection was performed with 100 μL of tetramethylbenzidine (1‐Step Ultra TMB ELISA; Pierce, Rockford, IL, USA) for 15 minutes. The reaction was stopped with 100 μL of HCl 1M and the optical density of each well was determined using a microplate reader set at 450 nm. Concentrations of Gla‐OCN and total OCN in the serum samples were calculated from polynomial second‐order standard curve obtained from the standard.

### Analyses of serum RANKL and SOST

Mouse RANKL and sclerostin (SOST) were measured in serum collected before or at 1 week after administration of DT, using the mouse RANKL ELISA kit (TNFSF11; ab100749) from ABCAM (Cambridge, UK) or the mouse/rat SOST ELISA kit (MSST00) from R&D Systems according to the manufacturers' instructions.

### Immunohistochemistry labeling of femoral bone sections

SOST protein was labeled in fixed and decalcified, paraffin‐embedded femoral bone sections as has previously been described^(^ ^19^ ^)^ (1:50; SOST primary antibody; biotinylated anti‐SOST [BAF1589]; R&D Systems).

### Metabolic cages

Female and male mice aged to 6 to 8 weeks were administered vehicle or DT on days 0, 2, 4, and 6. On day 7, mice were weighed and transferred to the Promethion Metabolic Cage System (Sable System, North Las Vegas, NV, USA) in the Physiology Core of MMCRI. Metabolic cage analysis was performed as has been previously described.^(^ ^21^ ^)^ Briefly, mice were acclimated in the cages for 24 hours prior to 72 hours of data collection with MetaScreen (Promethion, North Las Vegas, NV) v2.3.15.11. All data presented within are the 24‐hour averages of that 72‐hour period unless otherwise noted. All raw data generated for each mouse were processed using ExpeData version 1.9.27 (Sable System).^(^ ^21^ ^)^

### Statistical analysis

All data are expressed as mean ± SD on the mean unless otherwise noted. Student's *t* test, and ordinary one‐way or two‐way ANOVA tests were used to determine significance, using *p* < .05 as the cut‐off, with Tukey's multiple comparison post hoc testing unless otherwise noted. GraphPad Prism 6.02 software (GraphPad Software, La Jolla, CA, USA) was used to compute all statistical calculations unless otherwise noted. To query possible correlations between body composition and energy expenditure, ANCOVA analysis was performed using JMP software (SAS Institute, Cary, NC, USA).

## Results

### OCN+ cell depletion by DT treatment reduces trabecular bone in females and induces cartilage and mineralized tissue expansion of the primary spongiosa in males

**Fig 1:**
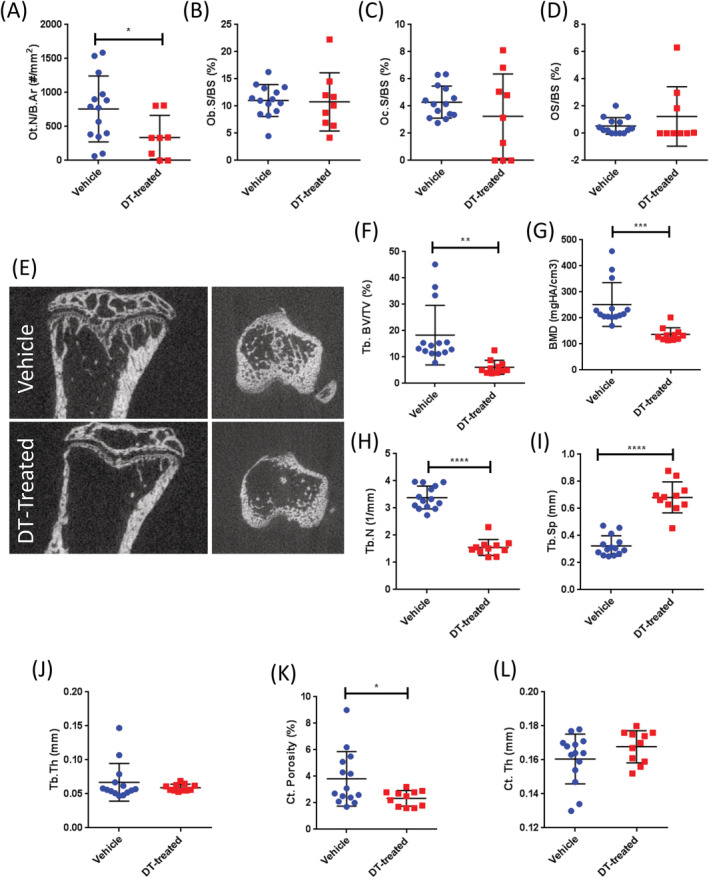
Diphtheria toxin (DT) treatment in osteocalcin‐driven Cre recombinase; inducible diphtheria toxin receptor (OCN‐Cre;iDTR) mice depletes osteocytes and reduces trabecular bone in female mice. (*A*) Quantification of bone via femoral static histomorphometry confirmed significantly reduced osteocyte numbers (osteocyte number per bone area [Ot.N/B.Ar], #/mm^2^). With no detectable effects on (*B*) osteoblast (Ob.S/BS,%), (*C*) osteoclast (Oc.S/BS,%), or (*D*) osteoid surface (OS/BS,%) per bone surface. Static histomorphometry data represent 8‐ to 10‐week‐old female mice: vehicle, *n* = 14; DT‐treated, *n* = 9. (*E*) Representative sagittal μCT images of tibial cross sections from vehicle (top) and DT‐treated (bottom) mice. Quantification of bone via tibial μCT revealed (*F*) significantly reduced trabecular bone volume per total volume (Tb.BV/TV, %), (*G*) reduced BMD, (*H*) fewer trabeculae (trabecular number [Tb.N], 1/mm), (*I*) with increased spacing (Tb.Sp, mm) and (*J*) no change in trabecular thickness (Tb.Th, mm). (*K*) Cortical porosity (%) was significantly reduced in DT‐treated females, (*L*) with no significant difference in cortical thickness (Ct. Th, mm). Tibial μCT data represent 8‐ to 10‐week‐old female mice: vehicle, *n* = 14; DT treated, *n* = 11. *p < .05; **p < .01; ***p < .001; ****p < .001 versus vehicle. Data shown as individual dot plots ± SD. All analyses were performed as a Student's *t* test with GraphPad Prism.

We began with a thorough examination of the bone phenotypes in DT‐treated OCN‐Cre;iDTR female mice by femoral histomorphometry. As expected, DT‐treated female mice exhibited significantly decreased numbers of osteocytes (Fig. 1*A*; Supplementary Fig. S1 *A*), confirming the targeting of OCN‐expressing cells. However, no significant effects on OBs were observed (Fig. 1*B*; Supplementary Fig. S1 *B*), perhaps because the total bone surface and bone perimeter were also decreased. DT treatment had no significant effect on osteoclast surface (Fig. 1*C*; Supplementary Fig. S1 *C*) or osteoid surface per bone surface (Fig. 1*D*) or on BMAT (Supplementary Fig. S1 *D*). Importantly, targeting osteocytes resulted in significantly reduced femoral trabeculae in overall volume and number (Supplementary Fig. S1 *E*,*F*) with increased trabecular spacing (Supplementary Fig. S1 *G*).

Female tibial bone parameters were then assessed by μCT (Fig. 1*E*; Supplementary Fig. S1 *H*); the tibial responses were consistent with the aforementioned femoral histomorphometry measurements. DT treatment altered the trabecular bone morphology in these female mice, as evidenced by significantly reduced trabecular bone volumes (Fig. 1*F*). Moreover, DT‐treated females exhibited significantly reduced trabecular BMD (Fig. 1*G*) and trabecular number (Fig. 1*H*), as well as increased trabecular spacing (Fig. 1*I*), with no significant change in trabecular thickness (Fig. 1*J*). Females also exhibited significantly reduced cortical porosity (Fig. 1*K*), with no significant change in cortical thickness (Fig. 1*L*) or cortical, total cross‐sectional, or marrow area (Supplementary Fig. S1 *I*–*K*). Collectively, these data suggest that DT treatment in female OCN‐Cre;iDTR mice results in reduced trabecular bone parameters with specific reduction of osteocytes.

**Fig 2:**
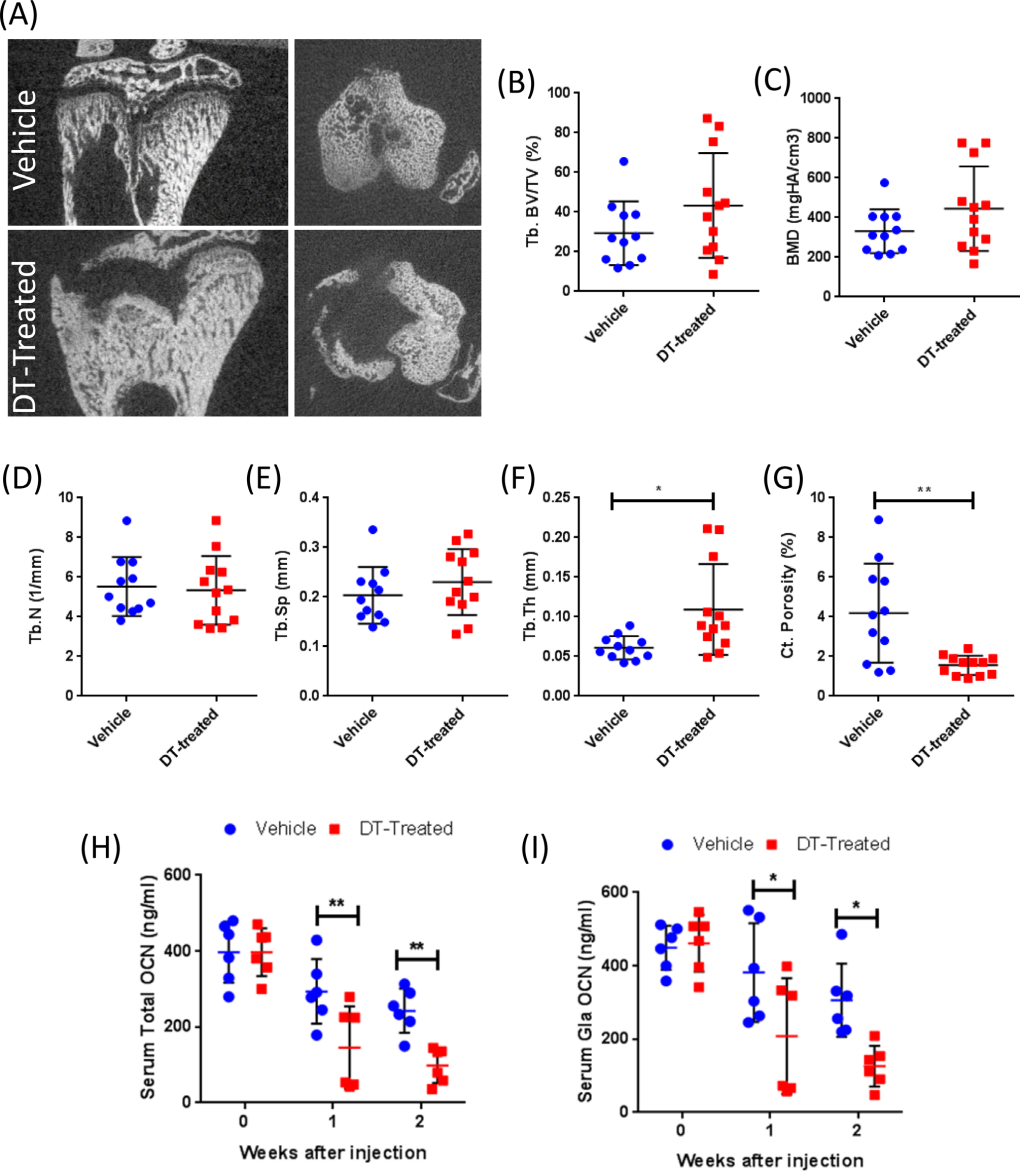
Diphtheria toxin (DT) treatment in osteocalcin‐driven Cre recombinase; inducible diphtheria toxin receptor (OCN‐Cre;iDTR) male mice induces varying effects on trabecular bone and the expansion of calcified primary spongiosa. (*A*) Representative sagittal μCT images of male tibias from vehicle (top) and DT‐treated (bottom). Quantification of bone via tibial μCT revealed no significant differences in (*B*) trabecular bone volume per total volume (Tb.BV/TV, %), (*C*) BMD, (*D*) trabecular number (Tb.N, 1/mm), or (*E*) trabecular spacing (Tb.Sp, mm). However, in male mice there was (*F*) a significant increase in trabecular thickness (Tb.Th, mm) and(*G*) reduced cortical porosity (%) in DT‐treated males. Tibial μCT data represent 8‐ to 10‐week‐old female mice: vehicle, *n* = 11; DT‐treated, *n* = 12. Reduced levels of serum total (*H*) and carboxylated (*I*) OCN as assessed via ELISA confirm the removal of OCN+ cells over time with DT injections (*n* = 6). *p < .05; **p < .01; ***p < .001; ****p < .001 versus vehicle. Data shown as individual dot plots ± SD. All analyses were performed as a Student's *t* test or one‐way ANOVA with GraphPad Prism.

Having observed the expected bone phenotype in female DT‐treated mice, we anticipated consistent loss of bone in both sexes; however, this was not the case for male DT‐treated OCN‐Cre;iDTR mice. In 9 of 12 DT‐treated males, we observed an abnormal phenotype within the primary spongiosa in the region of interest for histomorphometry, but because of this, the standard skeletal parameters, which are assessed adjacent to the femoral growth plate, could not be quantified. The three remaining mice exhibited no significant differences according to static histomorphometry (Supplementary Fig. S2*A*–*D*). DT treatment resulted in a wide variety of trabecular bone changes in these male mice, as can be seen in the tibial μCT images (Fig. 2*A*; Supplementary Fig. S2*E*,*F*), but there were no significant differences in trabecular bone volume (Fig. 2*B*), BMD (Fig. 2*C*), trabecular number (Fig. 2*D*), or trabecular spacing (Fig. 2*E*). Interestingly, we detected a significant increase in trabecular thickness (Fig. 2*F*), although this was varied. Consistent with the characterization in females, males also exhibited significantly reduced cortical porosity (Fig. 2*K*), with no differences in cortical, total cross‐sectional, or marrow areas (Supplementary Fig. S2*J*–*L*). These findings show a difference in the DT‐induced bone phenotype in OCN‐Cre;iDTR males compared with females.

To further investigate the abnormal phenotype observed in males, first we confirmed that OCN+ cells were being targeted by the DT treatment by performing an analysis of serum OCN levels throughout the duration of the DT treatment. We observed significantly lower levels of both total (Fig. 2*H*) and carboxylated (Fig. 2*I*) OCN in male DT‐treated mice, beginning after only 1 week of DT treatment and maximized at the end of the 2‐week injection period. Significantly reduced amounts of OCN confirmed that OCN‐producing cells were being targeted by our DT‐inducible model in males regardless of the absence of reduced bone.

**Fig 3:**
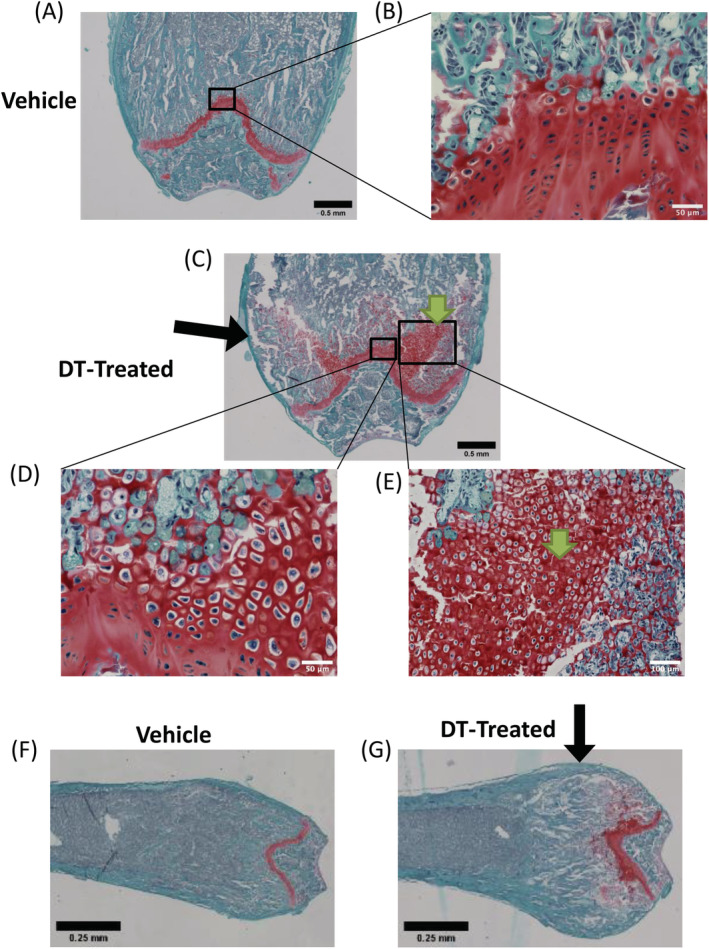
Diphtheria toxin‐ (DT‐) treated males showed hypertrophic chondrocytes with cartilage expansion into the primary spongiosa after osteocyte ablation. (*A*) Vehicle‐treated males (×4) showed normal safranin O staining (red), representative of cartilage, along the growth plate. (*B*) Zoomed‐in section (×40) along the growth plate showed relatively normal chondrocyte size in the vehicle‐treated males; scale bar = 50 μm. (*C*) DT‐treated males (×4) showed increased cartilage expansion into the primary spongiosa. (*D*) Zoomed‐in image (×40) along the growth plate showed hypertrophic chondrocytes along the growth plate; scale bar = 50 μm. (*E*) Zoomed‐in image (×20) showed increased cartilage expansion with the presence of hypertrophic chondrocytes throughout the primary spongiosa; scale bar = 100 μm. DT‐treated males exhibit an abnormal clubbing phenotype of the distal femur. Representative histology images of the vehicle‐treated males illustrate the normal distal femoral phenotype (*F*) compared with the DT‐treated males (*G*). Images were taken at ×4 magnification. Scale bar = 0.25 mm. Green arrows indicate abnormal region of hypertrophic chondrocytes and cartilage extending from the growth plate. Black arrows indicate a rounding or “clubbing” anatomic change in male femura.

We next more thoroughly characterized the bone phenotype observed in male mice by exploring if the changes in the growth plate observed in μCT could be related to cartilage. Safranin O staining of femoral sections confirmed normal trabecular bone and growth plate in vehicle‐treated males (Fig. 3*A*,*B*); however, DT‐treated male femurs contained large amounts of uncalcified hypertrophic chondrocytes and widened growth plates (Fig. 3*C*–*E*, Supplementary Fig. S3), as well as an expansion of calcified matrix, which was consistent with images and trends detected via tibial μCT. We also observed gross morphological phenotypic differences in DT‐treated males compared with vehicle‐treated with a rounding or “clubbing” of the distal femora (Fig. 3*F*,*G*), suggestive of osteopetrosis and a lack of RANKL in these mice. To specifically examine the hypothesis that RANKL was aberrantly expressed in this model, we assessed circulating RANKL levels in the serum of DT‐treated males after 1 week of treatment. We observed no significant differences in RANKL levels between the treatment groups by RANKL ELISA (Supplementary Fig. S3*D*).

In the females, we did not observe these effects (clubbing, hypertrophic chondrocytes, or expansion of calcified matrix), although safranin O staining did show some abnormalities at the growth plate, but to a much smaller extent than in males (Supplementary Fig. S4*A*). Von Kossa staining with safranin O counterstain confirmed the cartilage expansion originating from the growth plate was unmineralized, and there was an increase in mineralized trabecula‐like bone throughout the marrow cavity in DT‐treated male mice (Supplementary Fig. S4*B*). The increase in this disorganized, mineralized matrix and excess cartilage in the male mice is suggestive of increased chondrocyte differentiation followed by endochondral ossification, which seems to be occurring in males, but not females.

### OCN+ cell removal results in weight loss and alters serum lipid and blood chemistry

**Fig 4:**
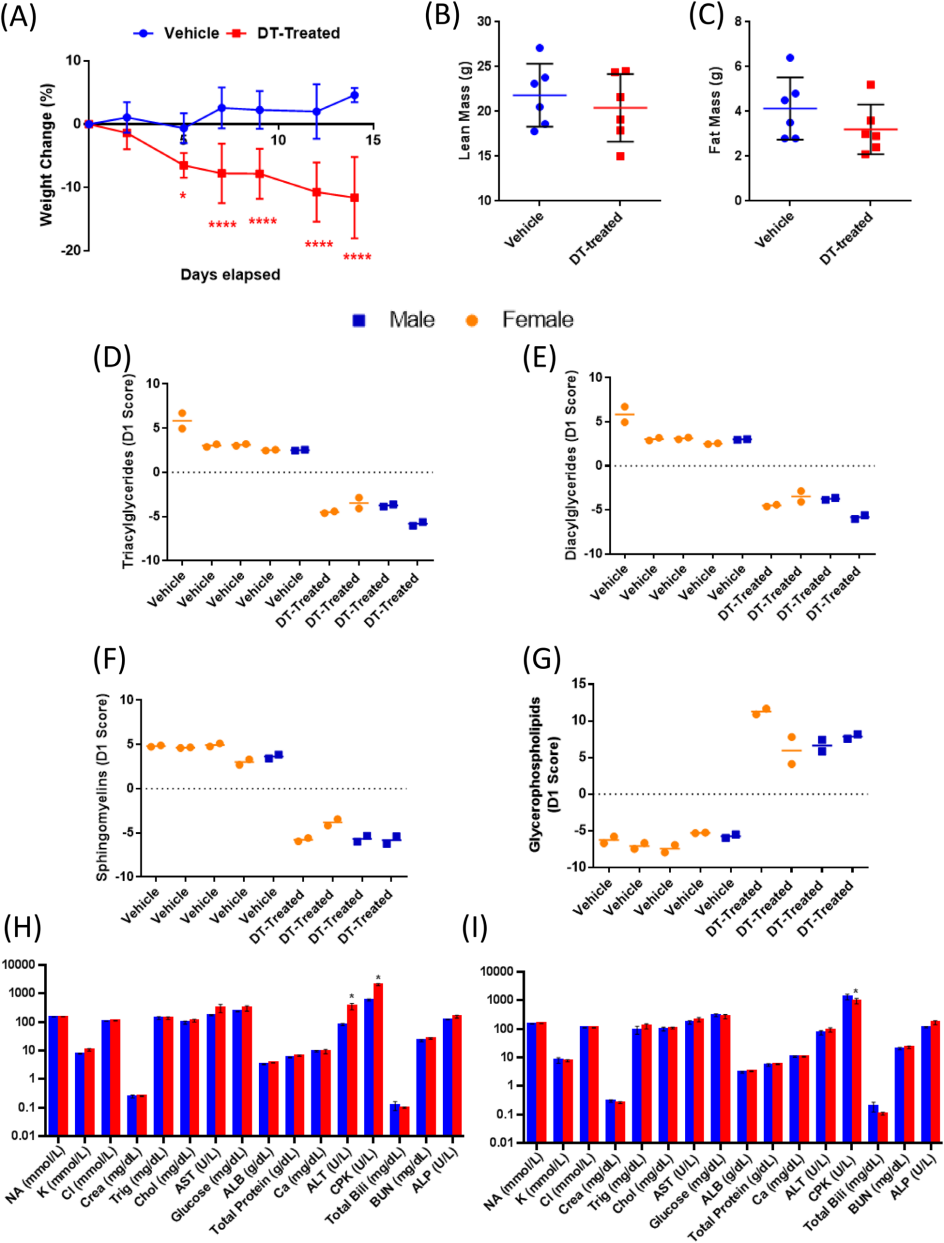
Diphtheria toxin‐ (DT‐) induced ablation of osteocytes results in rapid weight loss with moderate changes in body composition. (*A*) Weight change (%) in vehicle (*n* = 6) and DT‐treated (*n* = 6) males and females over the course of DT treatment. (*B*) Lean mass and (*C*) fat mass analyses via PIXImus in vehicle (*n* = 6) and DT‐treated (*n* = 6) mice at experimental day 14. DT‐induced bone loss results in significant changes in serum lipids and proteins. Lipidomic data are from blood serum taken at euthanization from mice treated with DT or vehicle, immediately after treatment (“No Recovery”) or after a 3‐week recovery phase (“Recovery”) from males and females. Principal component analysis of samples by lipid class: (*D*) Triacylglycerols, (*E*) diacylglycerols, (*F*) sphingomyelin, and (*G*) glycopeptidolipids. Osteocyte ablation presents sex‐specific effects in the muscular enzyme, creatinine phosphokinase (CPK), and the liver enzyme, alanine aminotransferase (ALT). (*H*) Female comprehensive chemistry panel. (*I*) Male comprehensive chemistry panel. *n* = 3 per group per sex. *p < .05 versus Vehicle. Data are shown as mean ± SD. All analyses were performed as 2‐way ANOVA + Sidak's multiple comparison tests with GraphPad Prism. ALB = Albumin Total Protein; ALP = Alkaline phosphatase; ALT = Alanine Aminotransferase; AST = Aspartate amino transferase Glucose; BUN = Blood urea nitrogen; Ca = Calcium; Chol = Cholesterol; Cl = Chloride; CPK = Creatine phosphokinase; Crea = Creatinine; K = Potassium; NA = Sodium; Total Bili = Total Bilirubin; Trig = Triglycerides

Although we observed sex‐related phenotypical differences in bone in response to DT, we observed a consistent effect on body composition in both sexes. Indeed, 2 weeks of DT treatment resulted in significant weight loss (Fig. 4*A*) in both females (Supplementary Fig. S5*A*) and males (Supplementary Fig. S5*B*), and nonsignificant trends toward reduced lean mass (Fig. 4*B*; Supplementary Fig. S5*C*,*D*) and fat mass (Fig. 4*C*; Supplementary Fig. S5*E*,*F*). These trends in body composition remained insignificant with normalization to body weight (data not shown). We next performed a large screen to characterize changes in circulating lipids (Table 1). Thirty‐six lipids were differentially represented in the sera of DT‐treated versus vehicle‐treated animals. Overall, there was a decrease in lipid storage species, including triacylglycerides (Fig. 4*D*) and diacylglycerides (Fig. 4*E*), as well as sphingomyelins (Fig. 4*F*), in response to DT, and a general increase in glycerophospholipids (Fig. 4*G*), including signaling lipids, specifically phosphatidylinositols.

**Table 1:** Top 10 Differentially Expressed Lipids in DT‐Treated Sera

Lipid peak name	*p* Value	DT to vehicle (fold‐change)
HexCer 40:1;2 (LCB 18:1;2‐2H2O,LCB 18:0;3‐3H2O)	2.82E‐05	0.533239958
GM1 42:4;2 (LCB 18:1;2‐2H2O,LCB 18:0;3‐3H2O)	.00018	0.587164266
PE 36:2 (FA 18:1)	.0011	10.37637816
PC 36:1;2 + HCOO (LPC pe)	.00291	2.026539759
CL 84:0 (PS,CL,PIP,PIP2,PIP3)	.00299	2.566257981
HexCer 40:2;3 (LCB 18:1;2‐2H2O,LCB 18:0;3‐3H2O)	.00467	0.549910319
CL 88:3 (FA 18:0)	.00585	2.364392893
HexCer 42:2;2 (LCB 18:1;2‐2H2O,LCB 18:0;3‐3H2O)	.00674	0.607829687
SM 32:2;3 (SM)	.00899	0.745250593
SM 42:1;2 (SM)	.00918	0.701738286

DT = Diphtheria toxin.

Having observed changes in serum lipids and in response to DT treatment, we used standard blood chemistry to monitor the overall health of the animals. Full blood chemistry analysis revealed varying creatine phosphokinase (CPK) levels in both females (Fig. 4*H*) and males (Fig. 4*I*) after targeting bone cells. In the females, CPK levels were significantly elevated in the DT‐treated mice compared with vehicle‐treated controls (Fig. 4*H*); however, CPK levels in males were significantly decreased (Fig. 4*I*). Females also showed an elevation of the liver enzyme, alanine aminotransferase (ALT; Fig. 4*I*); no such change was observed in males.

**Fig 5:**
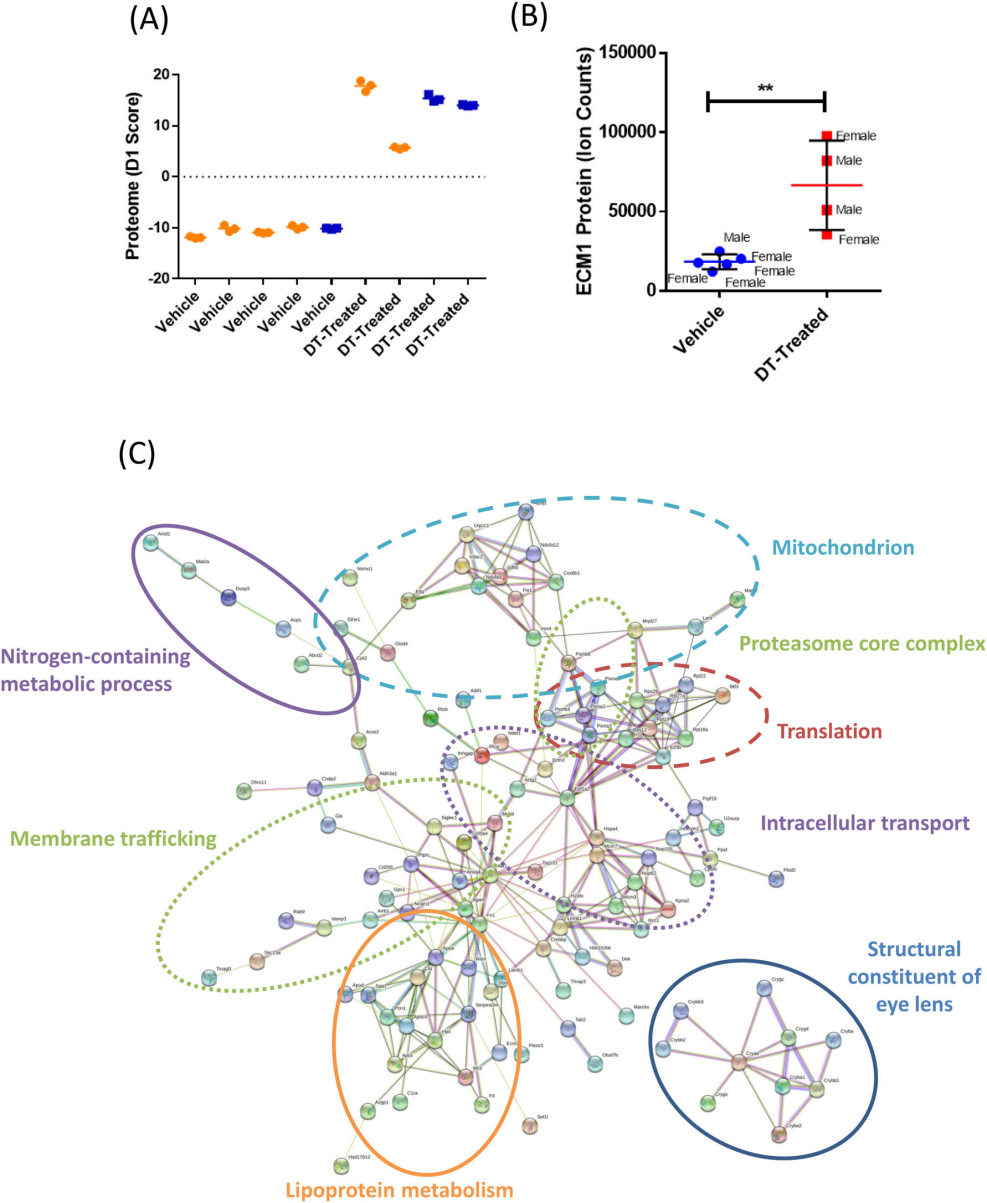
Osteocyte ablation results in significant changes in circulating protein levels and affects major homeostatic pathways. (*A*) Principal component analysis of proteomic data from blood serum of mice treated with vehicle or diphtheria toxin (DT). (*B*) Serum extracellular matrix protein.(*C*) Proteomic data from blood serum with 177 differentially expressed proteins could be delineated by String database analysis (https://string‐db.org/). *p < .05; **p < .01; ***p < .001; ****p < .001 versus vehicle. Data are shown as individual dot plots ± SD. All analyses were performed as a Student's *t* test or one‐way ANOVA with GraphPad Prism.

Serum proteomics revealed segregation of samples between vehicle‐treated and DT‐treated animals by PCA based on analysis of all detectable proteins (Fig. 5*A*; Table 2). In total, 392 proteins were significantly different (*p* < .05) between DT‐treated and vehicle‐treated animals, with 145 of them meeting the abundance cut‐off for proteomic analysis. Extracellular matrix protein 1 (ECM1) was elevated 3.62‐fold in DT‐treated animals (Fig. 5*B*; Table 2), whereas palatin‐like phospholipase domain‐containing protein 2 was significantly decreased in these samples (Table 2). Within the 145 aberrantly expressed proteins included in our analysis, 117 were connected in our network analysis (STRINGv11.0; Fig. 5*C*) with significant enrichment of proteins involved in critical processes such as membrane trafficking and intracellular transport and translation. Interestingly, we also observed enrichment of proteins associated with nitrogen‐containing metabolic processes and lipoprotein metabolism. These findings suggest that targeting bone cells in both sexes has similar systemic effects in modulating body composition and serum composition, but induces sexually dimorphic responses in skeletal phenotypes and blood enzymes.

**Table 2:** Top 10 Differentially Expressed Proteins in DT‐Treated Sera as Assessed by Proteomic Analysis

Dysregulated proteins	*p* Value	FC
Extracellular matrix protein 1	3.42E‐07	3.62
Inter‐alpha‐trypsin inhibitor heavy chain H3	3.68E‐07	2.25
Clusterin	4.44E‐06	2.22
Zinc‐alpha‐2‐glycoprotein O	1.45E‐05	3.69
Serine protease inhibitor A3M	2.73E‐05	3.07
Gelsolin	1.52E‐08	0.46
Patatin‐like phospholipase domain‐containing protein 2	.00024	0.40
Complement factor D	.00076	0.37
Very long‐chain specific acyl‐CoA dehydrogenase, mitochondrial	.00111	0.36
ADP‐ribosylation factor GTPase‐activating protein 3	.00168	0.37

DT = Diphtheria toxin; FC = _________.

### OCN+ cell depletion results in reductions in energy expenditure in mice

**Fig 6:**
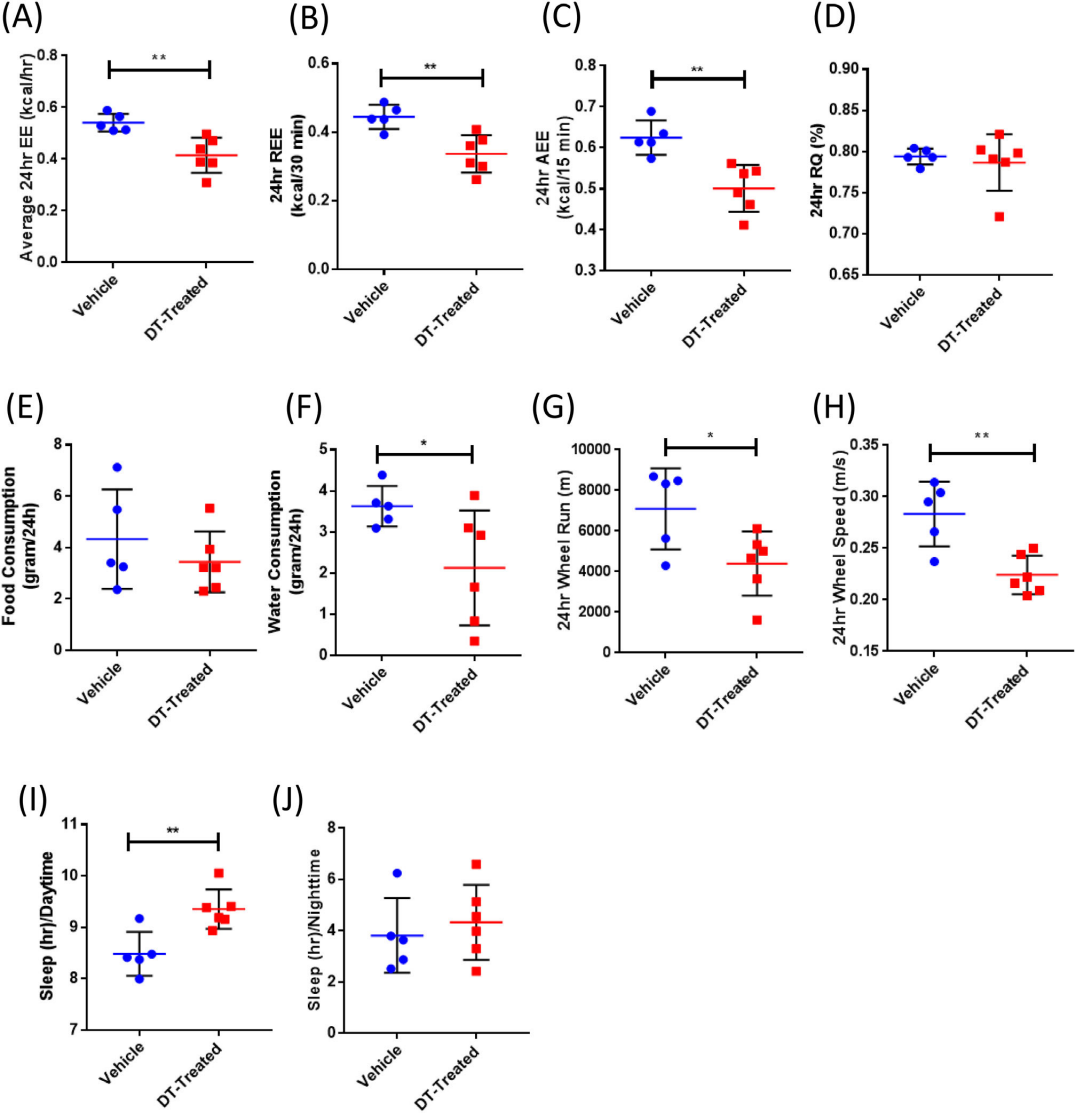
Diphtheria toxin‐ (DT‐) induced bone loss reduced female energy expenditure (EE). Metabolic cage analysis following 1‐week treatment of 6‐week‐old female mice (*n* = 5–6 females per group). (*A*) Average EE (kcal/h). (*B*) Resting energy expenditure (REE; kcal/30 min), (*C*) active energy expenditure (AEE; kcal/15 min), (*D*) respiratory quotient (RQ; %), (*E*) food consumption (g/24 h), (*F*) water consumption (g/24 h), (*G*) wheel distance (m) measured at walk or run, (*H*) wheel speed (m/s). Twelve‐hour‐cycle data for time spent sleeping (h) in the day (*I*) and night (*J*). *p < .05; **p < .01; ***p < .001; ****p < .001 versus vehicle. Data shown are individual dot plots ± SD. All analyses were performed as a Student's *t* test with GraphPad Prism.

To investigate the relationship between skeletal and overall systemic health, mice were subjected to metabolic cage analysis. DT‐treated females exhibited significant decreases in energy expenditure (EE; Fig. 6*A*). An ANCOVA analysis revealed lean mass to be a significant covariate (data not shown); however, the differences in EE remained significant when the variation in lean mass was taken into account between the two groups (Supplementary Fig. S5*C*,*D*). Resting energy expenditure (Fig. 6*B*) was also found to be reduced, suggesting the decreases in EE noted above were not caused by reductions in overall activity. Indeed, analysis of EE during levels of peak activity were found to be significantly lower as well (Fig. 6*C*), further indicating that the decreased EE noted in DT‐treated females was independent of activity. No significant changes in respiratory quotient or food consumption were noted (Fig. 6*D*,*E*); however, DT‐treated females consumed significantly less water across all cycles (Fig. 6*F*). In addition, females exhibited reductions in the distances run on the wheel (Fig. 6*G*) and the speed at which they ran (Fig. 6*H*) following DT treatment. Analysis of the 12‐hour cyclic data revealed that the greatest decrease in wheel activity was during the day (data not shown), and that this correlated with a significant increase in hours slept during this period (Fig. 6*I*,*J*).

**Fig 7:**
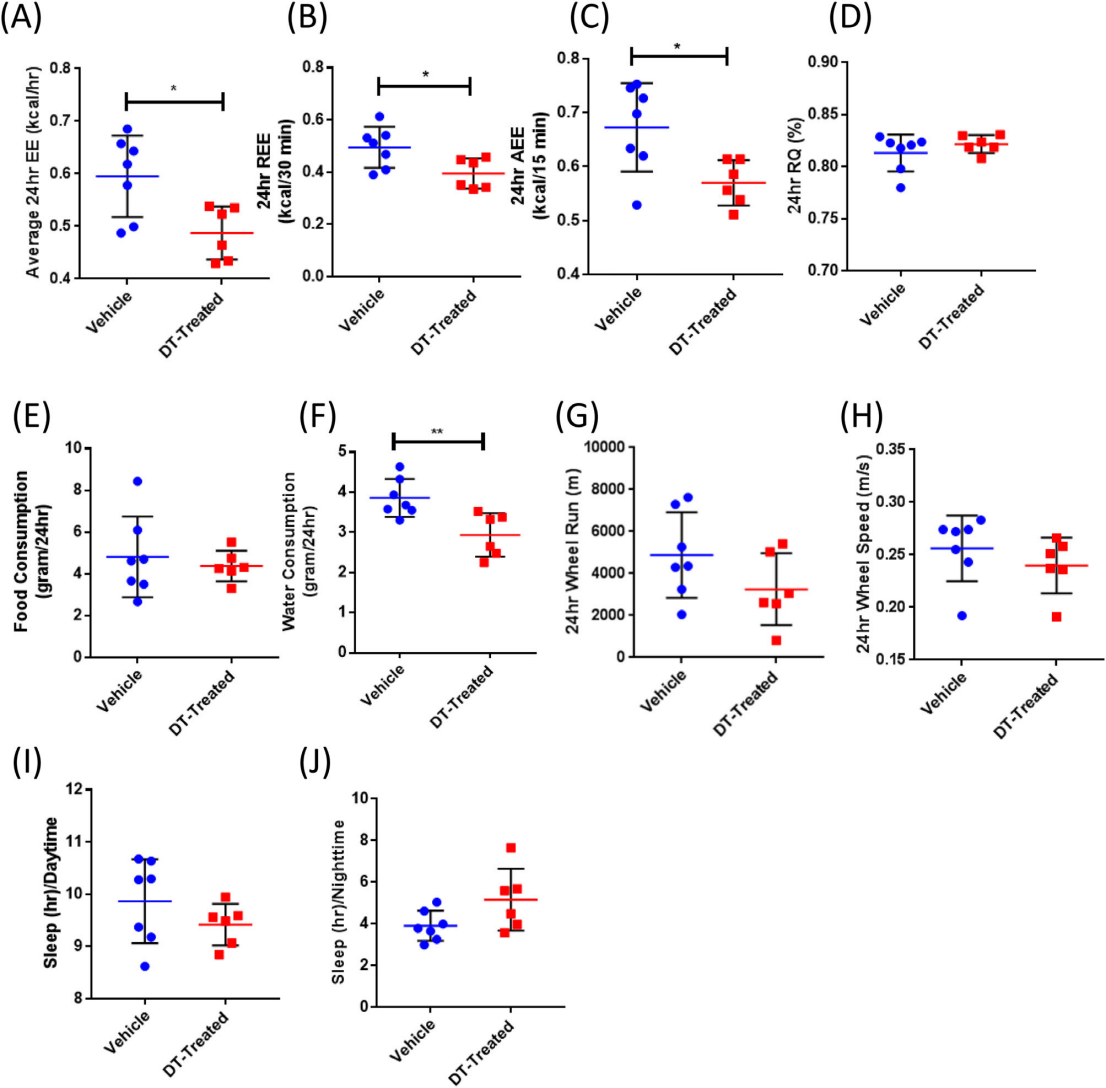
Diphtheria toxin‐ (DT‐) male mice had decreased energy expenditure (EE). Metabolic cage analysis following 1‐week DT treatment of 6‐week‐old male mice (*n* = 6–7 males per group). (*A*) Average energy expenditure (kcal/h), (*B*) resting energy expenditure (REE; kcal/30 min), (*C*) active energy expenditure (AEE; kcal/15 min), (*D*) respiratory quotient (RQ; %), (*E*) food consumption (g/24 h), (*F*) water consumption (g/day), (*G*) wheel distance (m) measured at walk or run, (*H*) wheel speed (m/s). Twelve‐hour‐cycle data for time spent sleeping (h) in the day (*I*) and night (*J*). *p < .05; **p < .01; ***p < .001; ****p < .001 versus vehicle. Data shown as individual dot plots ± SD. All analyses were performed as a Student's *t* test with GraphPad Prism.

Metabolic cage analysis of DT‐treated males revealed significant reductions in energy expenditure as was observed in females (Fig. 7*A*). Resting EE (Fig. 7*B*) and active EE were significantly lower (Fig. 7*C*) again, suggesting that the reductions in EE noted were independent of activity. No differences in overall respiratory quotient (Fig. 7*D*) or food consumption were observed (Fig. 7*E*); however, DT‐treated males, much like females, consumed significantly less water than controls (Fig. 7*F*). DT‐treated males, unlike females, exhibited no differences in wheel activity (Fig. 7*G*,*H*) or hours at rest (Fig. 7*I*,*J*). Importantly, the reductions in metabolism noted with DT treatment in OCN‐Cre;iDTR mice were not observed in DT‐treated C57BL/6J mice (Supplementary Fig. S6), supporting previous studies that the administration of DT has no effect on normal mice. We hypothesize that weight loss in the presence of reduced energy expenditure, with no significant differences in food intake, could be, in part, caused by reduced circulating SOST (based on data by Kim and colleagues),^(^ ^22^ ^)^ which we detected after 1 week of DT treatment in a separate, smaller cohort of male mice by serum ELISA (Supplementary Fig. S7*A*) and SOST immunohistochemistry of femoral histological sections (Supplementary Fig. S7*B*, *C*). Importantly these data further show that the metabolic phenotypes observed were specifically caused by bone cell depletion, implicating bone cells in the maintenance of normal energy metabolism.

## Discussion

In this study, we used the OCN‐Cre;iDTR model to deplete OCN+ cells (chondrocytes, OBs, and osteocytes) during a critical window of longitudinal growth. We observed sexually dimorphic bone phenotypes and decreased energy expenditure in both sexes in response to DT treatment. Interpreting such widespread complex changes in response to targeting OCN+ cells, underlines one of the limitations of this model, which includes targeting multiple cell types, making it difficult to speculate on a single mechanism driving our phenotypes. Nevertheless, our findings build on previously published work and further highlight bone as an endocrine organ.

Our results suggest that depletion of OCN+ cells with 2 weeks of DT treatment results in significantly reduced bone in young female mice through inhibiting bone formation, with no effect on resorption, consistent with Corral and colleagues.^(^ ^23^ ^)^ However, in males we observed no significant changes in bone parameters as measured by μCT, although further examination revealed expansion of unmineralized cartilage adjacent to the growth plate and a mineralized matrix tissue within the primary spongiosa. This response in females was similar to that seen in an osteocyte‐specific ablation model driven by a dentin matrix protein 1 promoter, which showed long‐term osteocyte deficiency resulted in substantial trabecular bone loss, as well as changes to trabecular microstructure.^(^ ^24^ ^)^

Surprisingly, the phenotype that we observed in male DT‐treated mice most closely resembled a number of conditional KO models for RANKL.^(^ ^25^ ^)^ Similar to what we observed, Xiong and colleagues found that when RANKL was deleted via OCN‐Cre in 5‐week‐old mice, expansion of the primary spongiosa and growth plates, as well as clubbing in the distal femur was observed. Moreover, osteocyte‐specific deletion of RANKL in adult mice led to an increase in bone mass,^(^ ^25^ ^)^ suggesting that osteocyte‐derived RANKL is responsible for the regulation of resorption and may contribute to the reduced cortical porosity that we observed in both sexes in our study. Although circulating RANKL was not significantly different in our male DT‐treated mice, it is possible that lower local concentrations of RANKL were deficient at the growth plate; hence, we believe the lack of RANKL remains a likely driver of the phenotypes we observed and requires further characterization.

The male DT‐treated mice had, to our surprise, an expansion of cartilage‐producing hypertrophic chondrocytes. It was once believed that terminally differentiated chondrocytes underwent apoptosis; however, previous studies have shown that during early‐stage development hypertrophic chondrocytes can transdifferentiate into OBs.^(^ ^26^ ^)^ These transdifferentiated OBs makeup a substantial proportion of the bone‐forming cells in mice.^(^ ^26^ ^)^ During late embryonic development and postnatal growth, mature chondrocytes maintain their ability to become OBs.^(^ ^26^ ^)^ Even at the late growth period of 11 weeks, chondrocytes contribute to the osteoblast pool within the primary spongiosa,^(^ ^26^ ^)^ and chondrocyte‐derived OBs also repair trabecular bone after insult, similar to developmental endochondral ossification involving a cartilage intermediate.^(^ ^22^, ^26^ ^)^ Thus, we propose that the increased safranin O staining and chondrocyte presence in males were a recovery response to the damage induced through the depletion of OCN+ cells.

In the growth plate, chondrocytes form columns that direct bone lengthening, a process that is driven by the rate of transition from proliferating chondrocytes to hypertrophic chondrocytes.^(^ ^4^ ^)^ In OCN‐Cre;iDTR mice, vehicle‐treated males exhibited relatively normal chondrocyte organization—with round “reserve” chondrocytes at the top of the growth plate—and clear columns of embedded chondrocytes at the growth plate. In DT‐treated males, but not females, we observed disorganized chondrocytes without clear columns and an expansion of the proliferative zone. We speculate that removal of bone‐forming cells during this critical developmental window creates this sexually dimorphic phenotype through a number of connected and complex mechanisms.

We are unsure what causes the sexual dimorphism we observed, but it is possible that during this period, elevated estrogen levels in females contribute to the restriction of the chondrocyte proliferative zone via ERα signaling.^(^ ^1^, ^2^ ^)^ Relatedly, androgen receptor (AR) mRNA has also been detected in proliferating and early hypertrophic chondrocytes in the growth plate of male and female rats, with males exhibiting higher AR mRNA and nuclear AR protein compared with females during sexual maturation.^(^ ^28^ ^)^ Combined, these studies indicate that chondrocytes are directly regulated by sex steroid hormones, which would be at their peak elevations during our DT‐treatment window. Additionally, growth hormone and IGF‐1 are heavily involved in postnatal growth and exhibit crosstalk with sex steroids during puberty. IGF‐1, which is higher in males than females during puberty, appears to be the primary determinant of skeletal sexual dimorphism, and likely interacts with both estrogens and androgens to elicit even greater differences in phenotype.^(^ ^1^, ^2^ ^)^ In the OCN‐Cre/*Igf1r* ^*flox/flox*^, it was reported that “abnormalities described… were qualitatively similar, but less pronounced in male experimental animals (data not shown),”^(^ ^29^ ^)^ suggesting that these mice may also have shown sexual dimorphism in response to removal of the IGF‐1 receptor on OCN+ cells. On the other hand, the phenotype we observed may be independent of sex hormones or insulin based on the work by Goring and colleagues, who found that conditional disruption of VEGF in OCN‐expressing cells in mice exerts a divergent influence on morphological, cellular, and whole‐bone properties between sexes; furthermore, they describe that the underlying sexual divergence in VEGF signaling in osteoblast cultures in vitro is independent of circulating sex hormones.^(^ ^10^ ^)^ Thus, it remains to be determined why we saw such differences between male and female bone phenotypes in response to ablation of OCN+ cells.

Prior publications describing the OCN‐Cre;iDTR mice have focused primarily on the important link between the skeleton and immune function. By specifically deleting OCN+ bone cells, these studies found defects in T‐cell lymphopoiesis^(^ ^10^ ^)^ and suppressed neutrophil response,^(^ ^11^ ^)^ along with a marked reduction in bone parameters. Work by Yu and colleagues used 4‐week‐old mice and found significantly reduced body size and body weight after 2 weeks of DT treatment, coupled with a decrease in bone volume, which is consistent with our results in females.^(^ ^10^ ^)^ These two prior studies did not examine or report on sexually dimorphic responses or any effects on chondrocytes.

DT‐treated mice exhibited changes in their blood sera lipid and protein profiles. In both males and females, there was a highly significant—nearly fourfold—increase in ECM1, which is expressed in many tissues including chondrocytes, and has been shown to regulate endochondral bone formation^(^ ^30^ ^)^ and angiogenesis.^(^ ^31^ ^)^ Overexpression of ECM1 via tissue‐specific transgene expression indicates that it inhibits both osteoblast and chondrocyte function; reduced bone parameters are seen in osteoblast‐specific overexpression models and growth plate abnormalities are seen in transgenic mice with chondrocyte‐specific overexpression.^(^ ^30^ ^)^ Future studies should examine whether elevated serum ECM1 contributes to the bone phenotypes we observed. Serum proteomics analysis also revealed that proteins involved in critical processes were aberrantly expressed upon depletion of bone cells (Fig. 5*C*), which lends strength to our finding that disrupting endochondral bone formation in developing mice has substantial effects on homeostasis.

OCN+ cell ablation also induced a few changes that were detectable in a standard blood chemistry panel including elevated CPK and ALT. CPK is a muscular enzyme that can be used to determine muscle deterioration or reduced muscular activity, and changes in CPK could support recent findings that bone and muscle exhibit crosstalk via OCN and RANKL.^(^ ^32^ ^)^ In particular, OCN has been characterized as a key regulator of muscle mass and exercise adaptation.^(^ ^7^, ^10^, ^18^, ^32^ ^)^ However, much of the prior research was performed with only in one sex, or the sex of the mice was not described, which may explain why the sexual dimorphism of OCN removal or OCN+ cell ablation was not described before. Female DT‐treated mice also showed an elevation of the liver enzyme ALT. Elevation of ALT could be coupled with the significant weight loss we observed because no other liver enzymes in the females were affected. Lack of additional significant findings in the full blood chemistry panel strengthens the specificity of the targeted ablation of bone cells as others have reported,^(^ ^10^, ^11^, ^24^, ^28^ ^)^ because no signs of organ damage were detected.

Here, we demonstrate that targeting bone cells has an effect on animal metabolism and overall health. Our metabolic cage analyses revealed significantly decreased energy expenditure in both sexes following DT treatment. By targeting OCN+ cells, DT treatment is likely altering OBs and osteocytes, which secrete additional hormones involved in the control of energy metabolism.^(^ ^33^, ^34^ ^)^ This could explain the complex metabolic changes observed in our model. Depletion of OCN alone, the osteoblast‐derived hormone that signals to various organs throughout the body and plays a role in multiple energy‐related processes,^(^ ^6^, ^7^, ^9^ ^)^ likely contributes to the reduced energy expenditure phenotype we observed. Overall, our data support the notion that OCN+ cells are crucial regulators of whole‐body energy metabolism and bone‐building activities during development, and that removal of these cells and their signaling factors has different effects based on the organism's sex.

## Author Contributions

**Heather Fairfield:** Conceptualization; data curation; formal analysis; investigation; methodology; project administration; resources; software; supervision; validation; visualization; writing‐original draft; writing‐review and editing. **Samantha Costa:** Data curation; investigation; methodology; resources; visualization; writing‐review and editing. **Victoria DeMambro:** Data curation; formal analysis; investigation; software; writing‐original draft; writing‐review and editing. **Celine Schott:** Formal analysis; methodology; resources; writing‐review and editing. **Janaina Martins:** Formal analysis; methodology; visualization; writing‐review and editing. **Mathieu Ferron:** Conceptualization; data curation; funding acquisition; investigation; methodology; resources; supervision. **Calvin Vary:** Data curation; investigation; methodology; resources; software; validation; visualization; writing‐original draft; writing‐review and editing.

## Authors' roles

Study design: HF and MR. Study conduct: HF, SC, and MR. Data collection: MR, HF, SC, and JM. Data analysis: HF, SC, JM, and MR. Metabolic cage experiments: VD. Data interpretation: MR, CV, HF, VD, SC, CS, and MF. Serum analyses: HF, SC, CV, CS, and MF. Drafting manuscript: HF, SC, and MR. Revising manuscript content: HF, SC, VD, MF, and MR. Approving final version of manuscript: MR, HF, CV, SC, CS, MF, and JM. MR takes responsibility for the integrity of the data analysis.

## Supporting information


**Supplementary Figure S1 The effects of DT‐treatment in OCN‐Cre;iDTR female long bones.** Femoral histomorphometry confirmed reduced osteocyte numbers per total area (Ot.N/T.AR, #/mm^2^) (A), with no significant differences in osteoblast number (Ob.N/B.pm, #/mm) (B), or osteoclast number (Oc.N/B.pm, #/mm) (C) per bone perimeter, or in bone marrow adipose volume per total volume (AV/TV, %) (D). Trabecular bone volume per total volume (Tb. BV/TV, %) (E), trabecular number (Tb.N, 1/mm) (F) and trabecular spacing (Tb.Sp, mm) (G) were all significantly affected by DT treatment in female mice. Cortical area (I), total cross‐sectional area (J) and marrow area (K) as assessed by tibial μCT were unchanged with DT‐treatment. Static histomorphometry data represents 8‐10‐week‐old female mice, vehicle‐treated n = 14, DT‐treated n = 9. Additional representative μCT images of tibial cross sections (H) from vehicle‐treated (top) and DT‐Treated (bottom). **** *p* < .001; *** *p* < .001; ** *p* < .01; * *p* < .05 vs Vehicle‐treated. Data shown as individual dot plots ± S.D. All analyses were performed as a Student's T‐test within Prism.
**Supplementary Figure S2. The effects of DT‐treatment in OCN‐Cre;iDTR male long bones.** Femoral histomorphometry revealed no effect on osteocyte numbers per total area (Ot.N/T.AR, #/mm^2^) (A), and no significant differences in either osteoblast surface (Ob.S/BS, %) (B) or osteoclast surface (Oc.S/BS, %) (C) per bone surface, or osteoid surface per bone surface (OS/BS, %) (D). Static histomorphometry data represents 8‐10‐week‐old male mice, vehicle‐treated n = 8, DT‐treated n = 3. Additional representative μCT images of tibial cross sections (E, F) from vehicle‐treated (top) and DT‐Treated (bottom). Cortical area (J), total cross‐sectional area (K) and marrow area (L) as assessed by tibial μCT were unchanged with DT‐treatment. Tibial μCT data represents 8‐10‐week‐old male mice. Data shown as individual dot plots ± S.D.
**Supplementary Figure S3. Cartilage expansion in the presence of hypertrophic chondrocytes was characterized in the majority of OCN‐Cre;iDTR male mice treated with DT.** Representative safranin O (red) stained image of DT‐treated male (4x) showed extensive cartilage expansion into the femoral primary spongiosa (A). Zoomed in section (20x) along the growth plate highlighting hypertrophic chondrocytes (B), Scale bar = 100 μm. Four representative images (4x) of DT‐treated males with increased cartilage expansion from the growth plate into the primary spongiosa with evidence of further femoral clubbing, as previously shown in Figure 3 (C). Scale bar = 0.5 mm. Green arrows indicate abnormal region of hypertrophic chondrocytes and cartilage extending from the growth plate observed in males. Black arrows indicate a rounding or “clubbing” anatomic change in male femora. Serum RANKL (D) as measured by ELISA in vehicle‐treated (n = 8) and DT‐treated (n = 6) male mice.
**Supplementary Figure S4. Chondrocyte expansion of the femur after DT‐treatment proved to be sex‐specific and non‐mineralized cartilage.** Vehicle‐treated females and males (left panel) showed normal cartilage staining (Safranin O, red) along the growth plate (A), while DT‐treated males (lower right panel) showed an expansion of cartilage that was not observed in the DT‐treated females (upper right panel) (A). Representative femoral histology images with von Kossa and Safranin O counterstain in DT‐treated males showed cartilage expansion stemming from the growth plate was not mineralized, yet there was an increase in mineralized trabecular bone beyond the primary spongiosa (B). Images were taken at 4x magnification. Scale bar = 0.5 mm. White arrows indicate region of expansion of calcified matrix, only observed in male mice.
**Supplementary Figure S5. Body composition analysis of 8‐week old female and male mice after 2 weeks of DT‐treatment.** Weight change (%) in females (A) and males (B) throughout DT‐treatment (n = 3 females, n = 3 males) compared to vehicle‐treated (n = 3 females, n = 3 males; PIXI analysis (n = 3 per group per sex) of lean mass (g) in females (C) and males (D), fat mass (g) in females (E), and males (F). The ratio of fat mass/tissue mass (%) in females (G) and males (H). n = 3. **** *p* < .001; *** *p* < .001; ** *p* < 0.01; * *p* < 0.05 vs Vehicle‐treated. Data are shown as individual dot plots ± S.D. All analyses were performed as a Student's T‐test within Prism.
**Supplementary Figure S6. DT‐treatment in C57BL/6J mice shows no effect on metabolic activity.** Metabolic cage analysis was conducted following one‐week of DT‐treatment in 8‐week old mice; n = 5 per group. Average energy expenditure (EE) (kcal/h) (A), resting energy expenditure (REE) (kcal/30 min) (B), active energy expenditure (AEE) (kcal/15 min) (C), respiratory quotient (RQ, %) (D), food consumption (g/24 h) (E), water consumption (g/day) (F), wheel distance (m/24 h) (G), wheel speed (m/s) (H). 12 hour cycle data for time spent sleeping (h) in the day (I) and night. **** *p* < .001; *** *p* < .001; ** *p* < .01; * *p* < .05 vs Vehicle‐treated. Data are shown as individual dot plots ± S.D. All analyses were performed as a Student's T‐test within Prism.
**Supplementary Figure S7. Decreased sclerostin levels could contribute to body weight reductions in DT‐treated OCN‐Cre;iDTR mice**. Osteocyte‐secreted SOST is significantly decreased in circulation (blood serum) by day 7 of DT treatment as measured via rat/mouse ELISA (A); Vehicle‐treated (n = 3), DT‐treated (n = 3), male mice aged 6 weeks at the start of treatments. Sclerostin immunohistochemistry effectively labeled osteocytes in vehicle treated animals (B) while the majority of osteocytes had been removed by 14 days of DT treatment (C), leaving empty canaliculi and very little SOST label (arrows); images of cortical bone from proximal femur taken at 40X; mice aged 6–8 weeks at the start of treatments.

**Appendix S1**. Supporting Information.
