# Supplementary material for: Targeting Bone Cells During Sexual Maturation Reveals Sexually Dimorphic Regulation of Endochondral Ossification
Source: JBMR Plus. 2020 Oct 14;4(11):e10413. doi: 10.1002/jbm4.10413 (PMC7657395; doi:10.1002/jbm4.10413)
Supplement: Supplementary file 1 — Supplementary Figure S1 The effects of DT‐treatment in OCN‐Cre;iDTR female long bones. Femoral histomorphometry confirmed reduced osteocyte numbers per total area (Ot.N/T.AR, #/mm2) (A), with no significant differences in osteoblast number (Ob.N/B.pm, #/mm) (B), or osteoclast number (Oc.N/B.pm, #/mm) (C) per bone perimeter, or in bone marrow adipose volume per total volume (AV/TV, %) (D). Trabecular bone volume per total volume (Tb. BV/TV, %) (E), trabecular number (Tb.N, 1/mm) (F) and trabecular spacing (Tb.Sp, mm) (G) were all significantly affected by DT treatment in female mice. Cortical area (I), total cross‐sectional area (J) and marrow area (K) as assessed by tibial μCT were unchanged with DT‐treatment. Static histomorphometry data represents 8‐10‐week‐old female mice, vehicle‐treated n = 14, DT‐treated n = 9. Additional representative μCT images of tibial cross sections (H) from vehicle‐treated (top) and DT‐Treated (bottom). **** p < .001; *** p < .001; ** p < .01; * p < .05 vs Vehicle‐treated. Data shown as individual dot plots ± S.D. All analyses were performed as a Student's T‐test within Prism. Supplementary Figure S2. The effects of DT‐treatment in OCN‐Cre;iDTR male long bones. Femoral histomorphometry revealed no effect on osteocyte numbers per total area (Ot.N/T.AR, #/mm2) (A), and no significant differences in either osteoblast surface (Ob.S/BS, %) (B) or osteoclast surface (Oc.S/BS, %) (C) per bone surface, or osteoid surface per bone surface (OS/BS, %) (D). Static histomorphometry data represents 8‐10‐week‐old male mice, vehicle‐treated n = 8, DT‐treated n = 3. Additional representative μCT images of tibial cross sections (E, F) from vehicle‐treated (top) and DT‐Treated (bottom). Cortical area (J), total cross‐sectional area (K) and marrow area (L) as assessed by tibial μCT were unchanged with DT‐treatment. Tibial μCT data represents 8‐10‐week‐old male mice. Data shown as individual dot plots ± S.D. Supplementary Figure S3. Cartilage expansion in t [file JBM4-4-e10413-s001.pptx]

## Slide 1
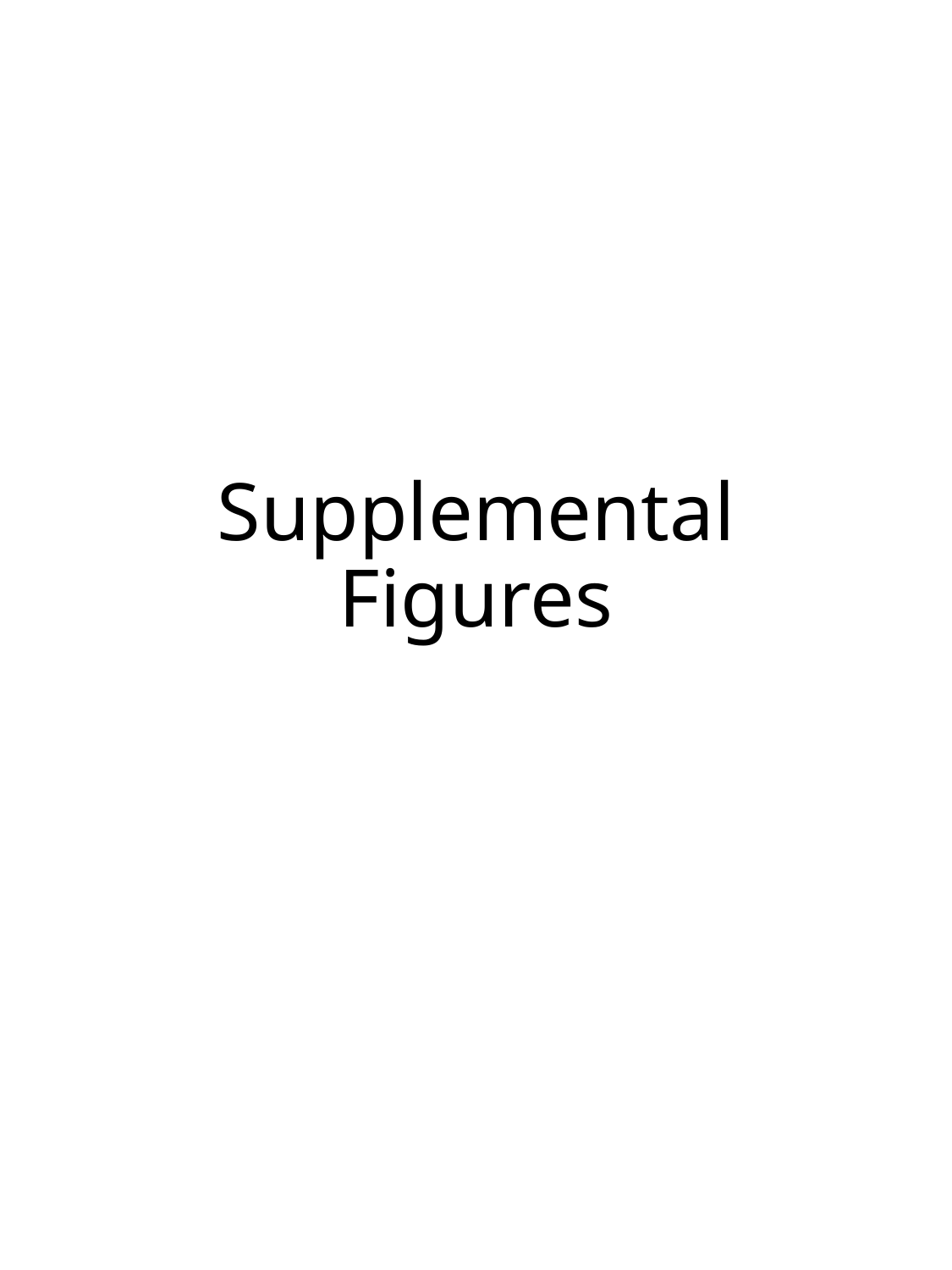

# Supplemental Figures

## Slide 2
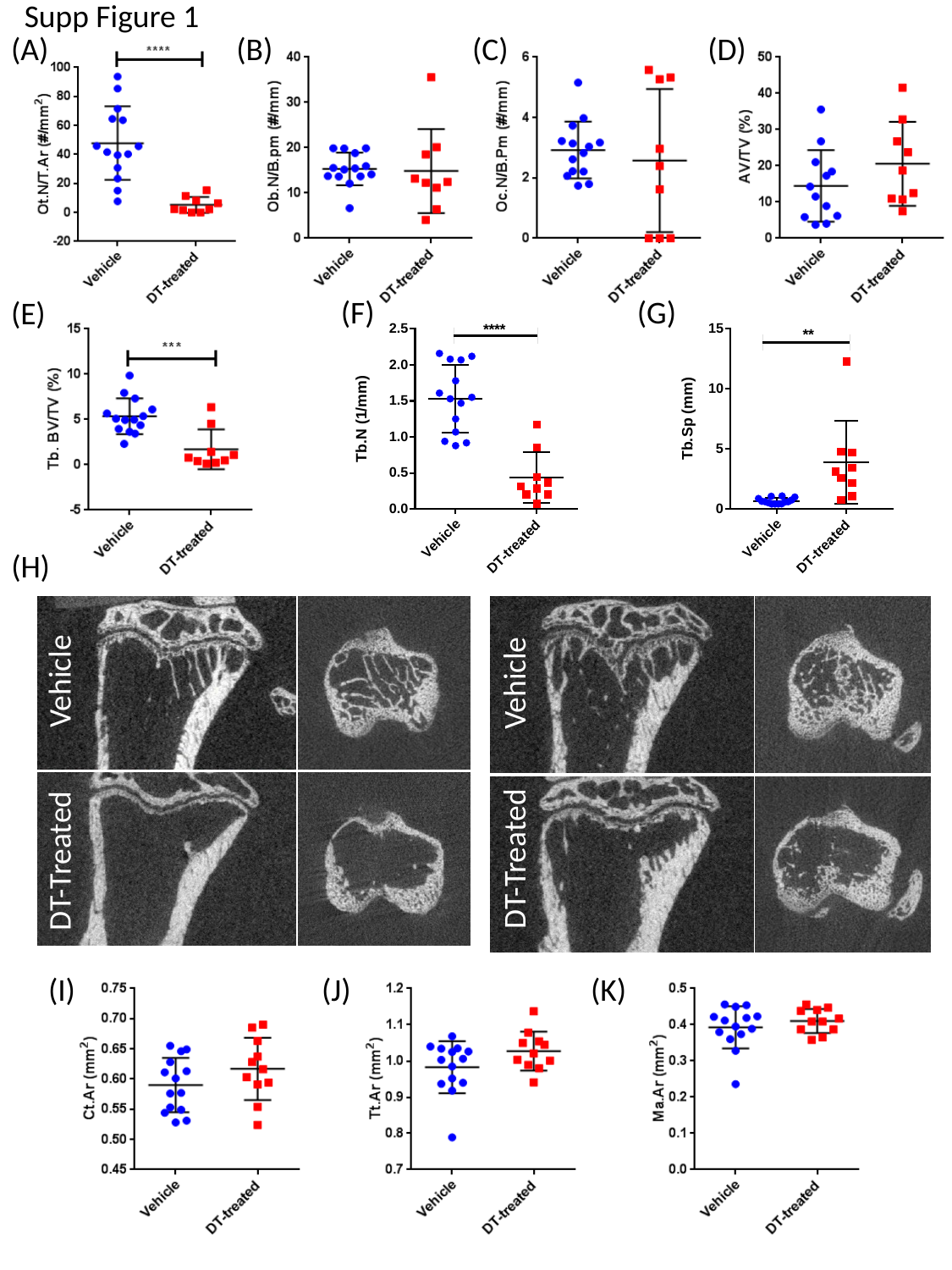

Supp Figure 1
(C)
(D)
(A)
(B)
(F)
(G)
(E)
(H)
Vehicle
DT-Treated
Vehicle
DT-Treated
(I)
(J)
(K)

## Slide 3
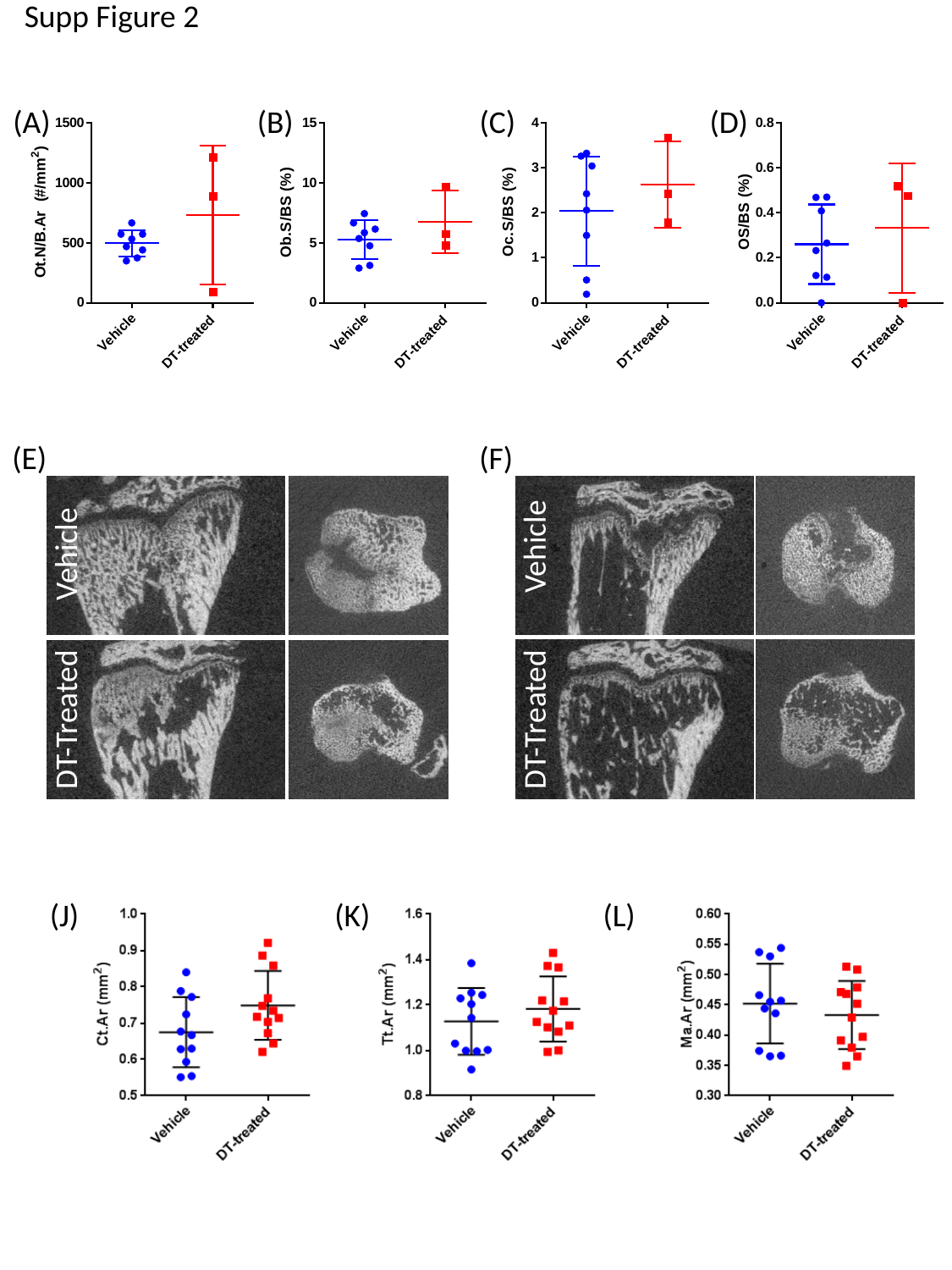

Supp Figure 2
(A)
(B)
(C)
(D)
(E)
(F)
Vehicle
DT-Treated
Vehicle
DT-Treated
(J)
(K)
(L)

## Slide 4
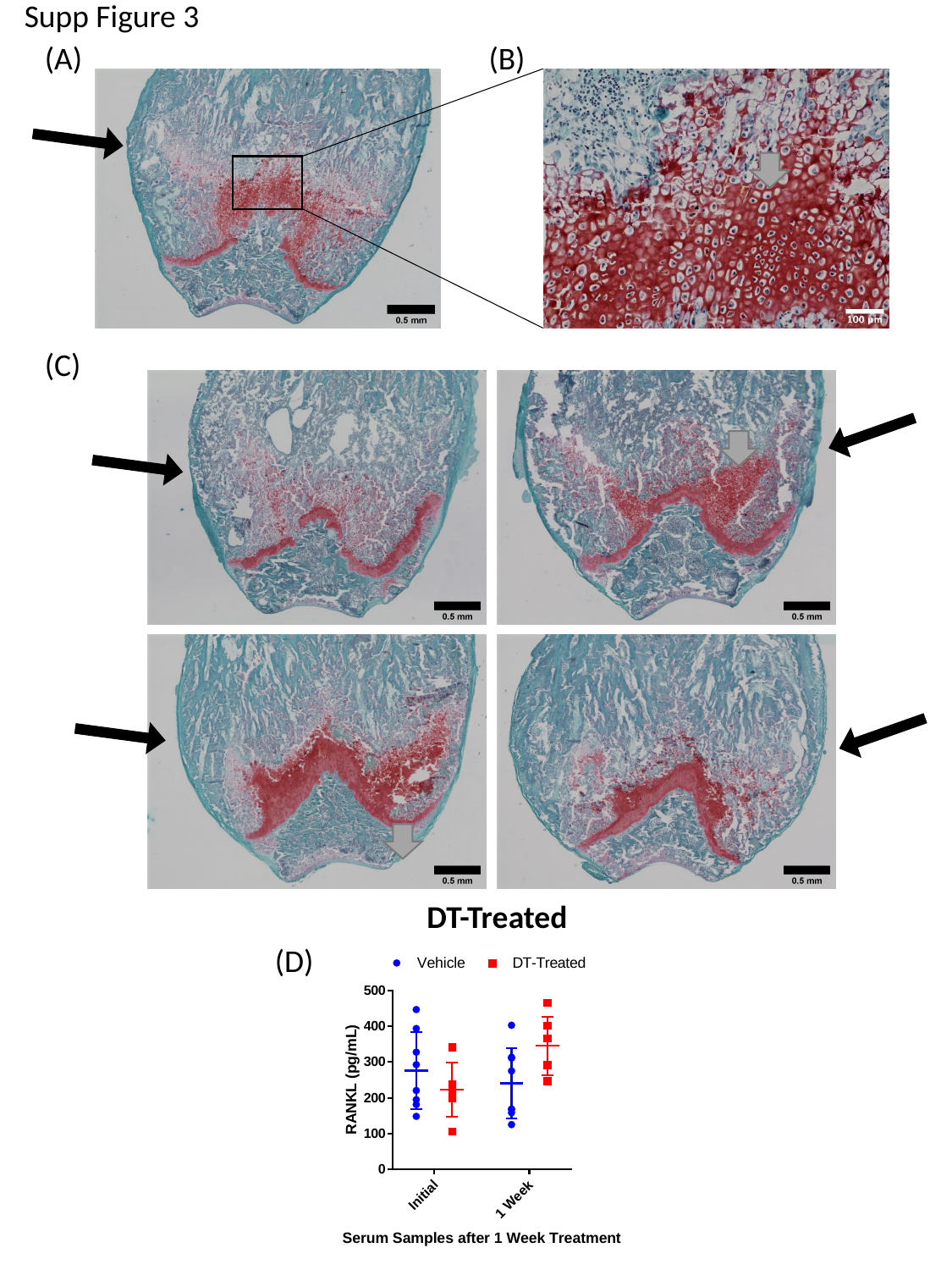

Supp Figure 3
(A)
(B)
(C)
DT-Treated
(D)

## Slide 5
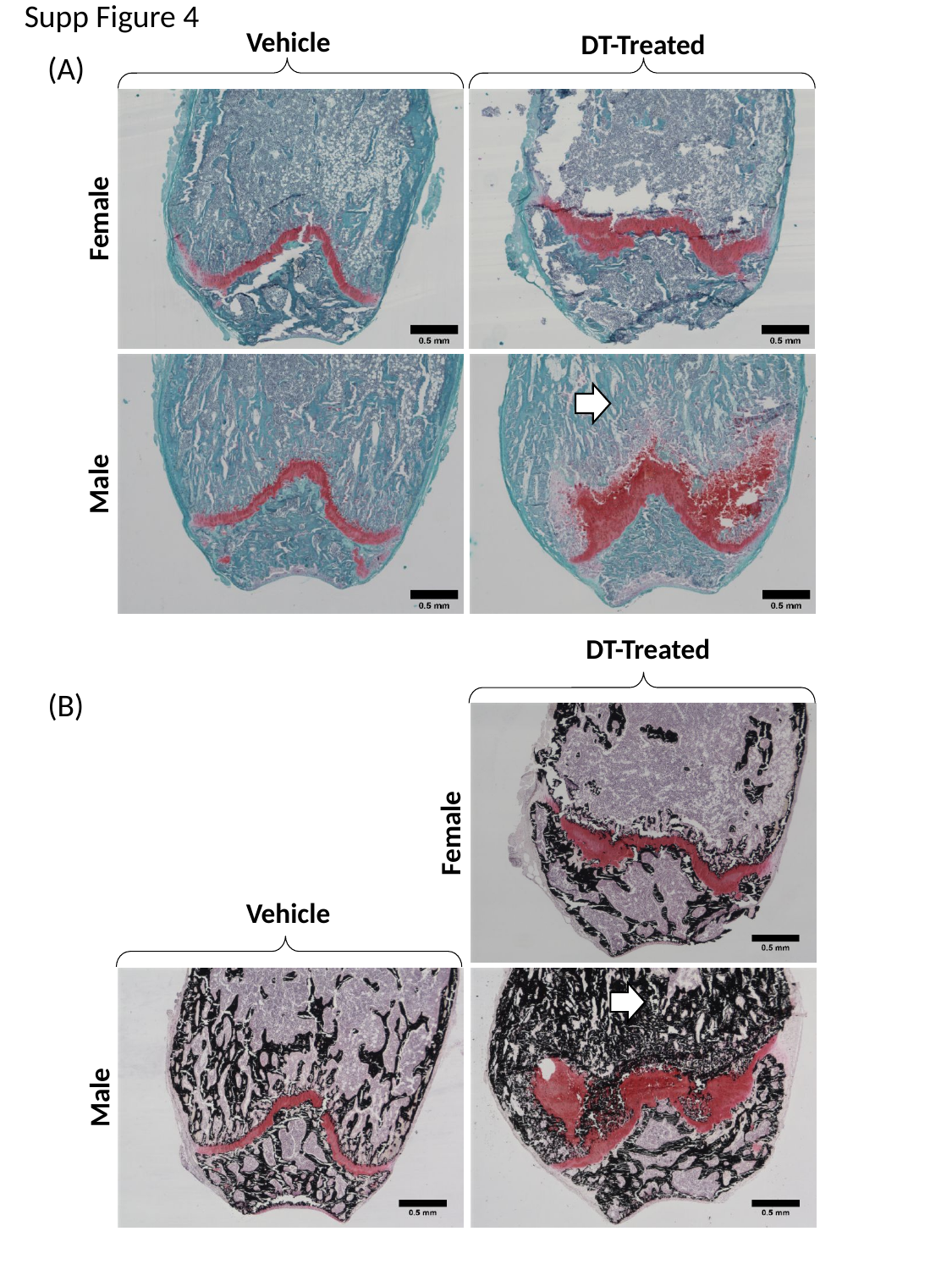

Supp Figure 4
Vehicle
DT-Treated
Female
Male
(A)
DT-Treated
Female
Vehicle
Male
(B)

## Slide 6
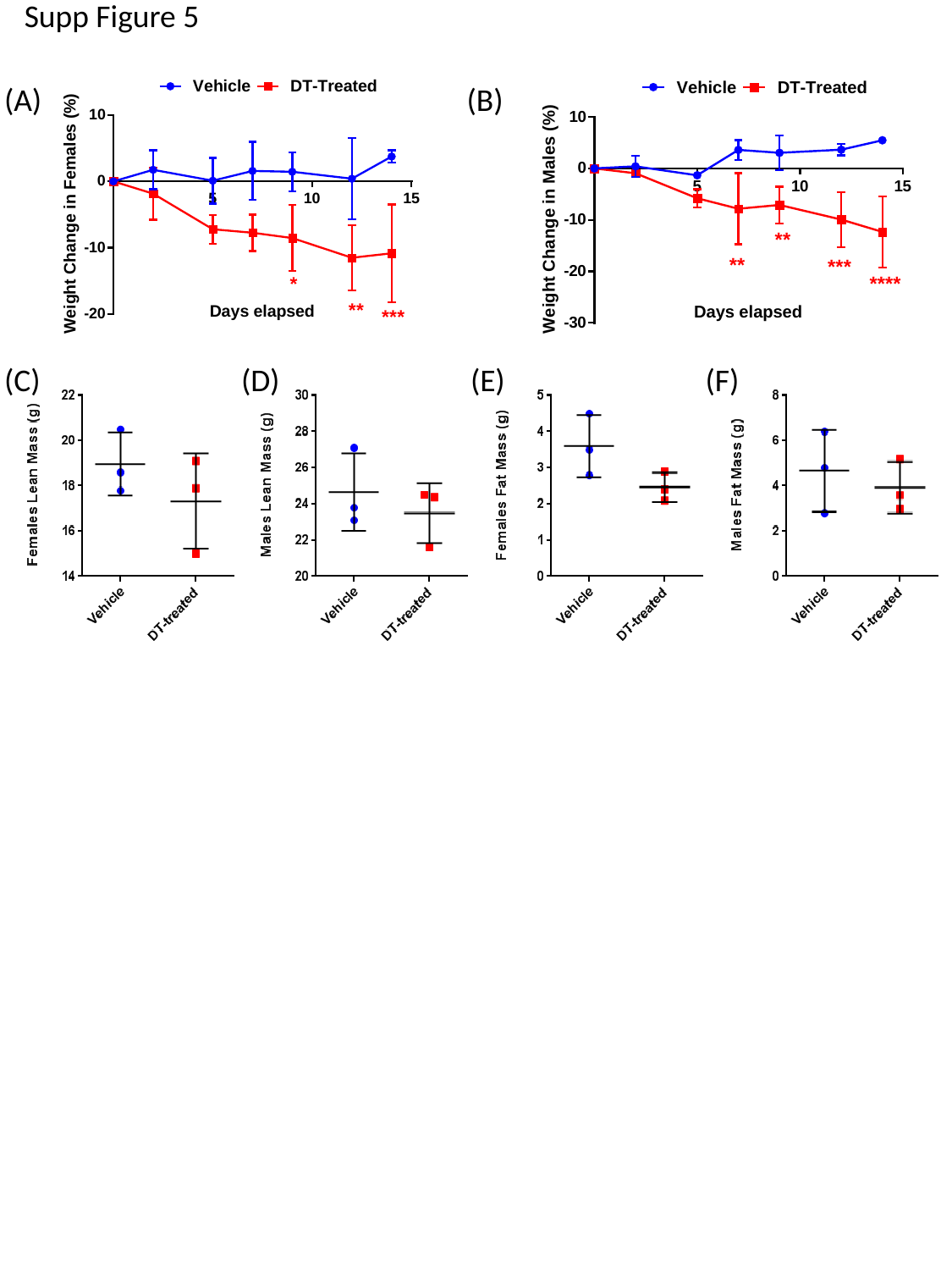

Supp Figure 5
(A)
(B)
(C)
(D)
(E)
(F)

## Slide 7
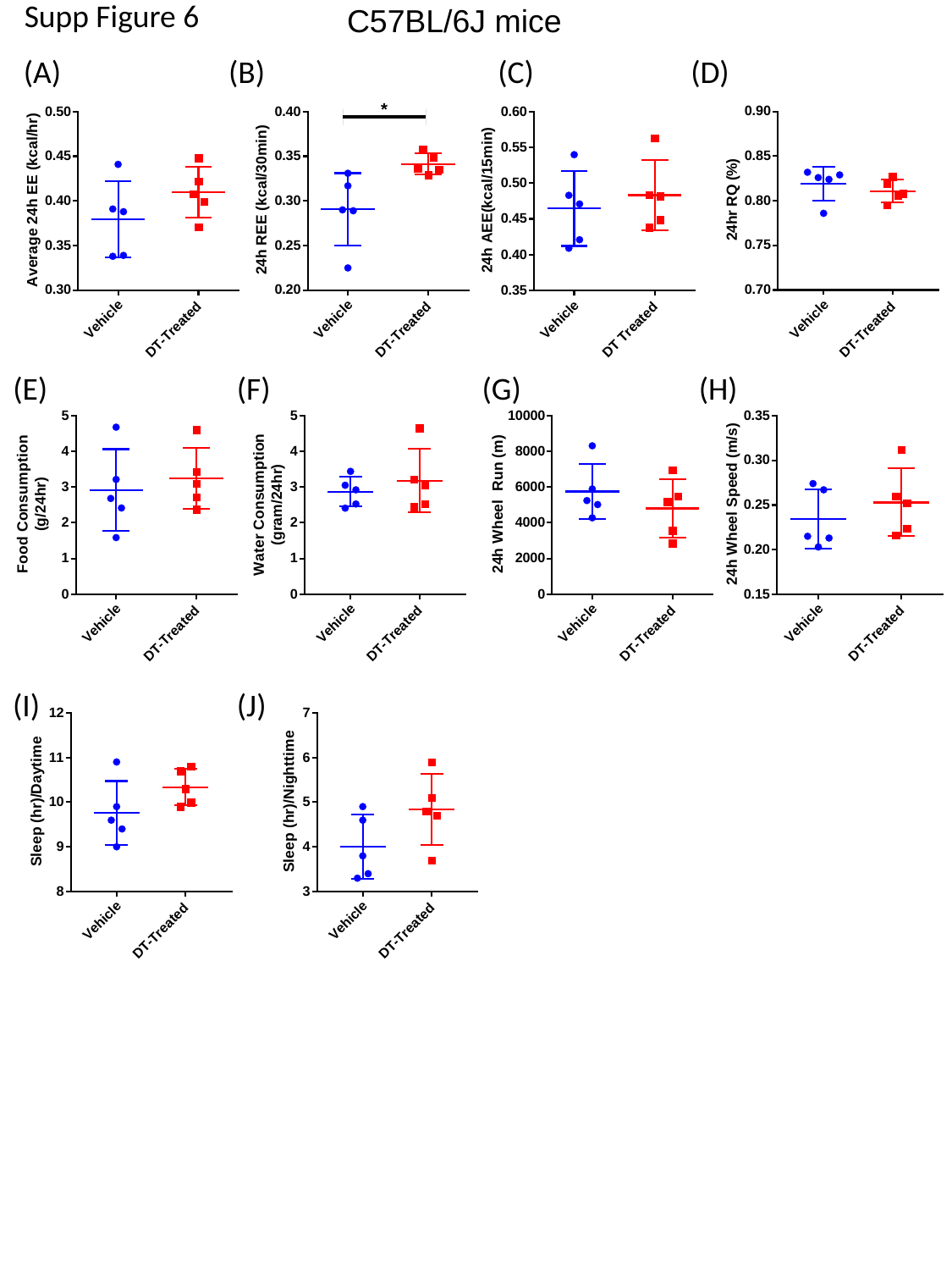

Supp Figure 6
C57BL/6J mice
(A)
(B)
(C)
(D)
(E)
(F)
(G)
(H)
(I)
(J)

## Slide 8
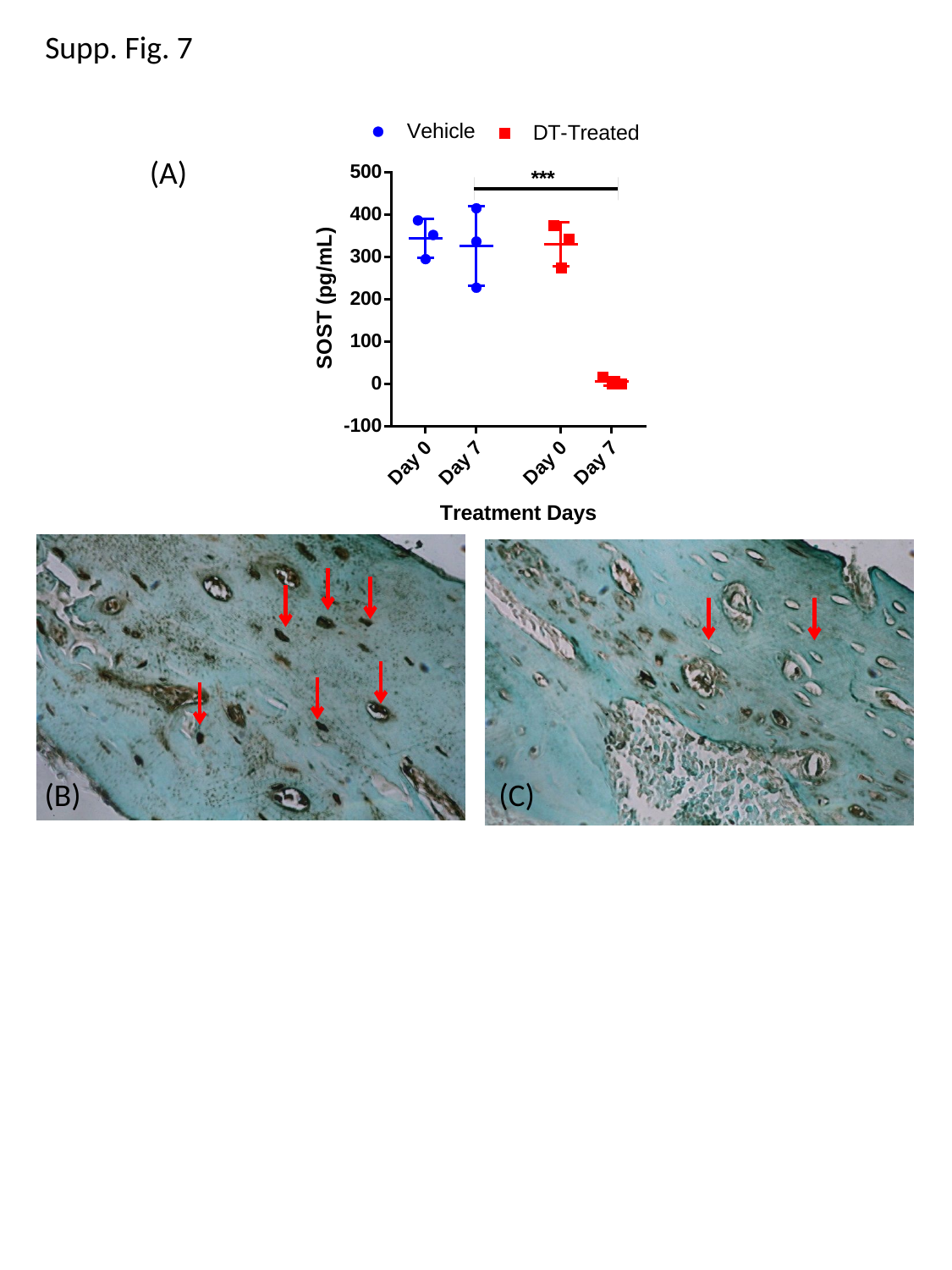

Supp. Fig. 7
(A)
(B)
(C)
