# Supplementary material for: Targeting Bone Cells During Sexual Maturation Reveals Sexually Dimorphic Regulation of Endochondral Ossification
Source: JBMR Plus. 2020 Oct 14;4(11):e10413. doi: 10.1002/jbm4.10413 (PMC7657395; doi:10.1002/jbm4.10413)
Supplement: Supplementary file 2 — Appendix S1. Supporting Information. [file JBM4-4-e10413-s002.docx]

**Supplemental Methods**

**Microcomputed tomography:** Tibiae were imaged using a µCT scanner (µCT-40) (Scanco Medical, Bruttisellen, Switzerland). Trabecular bone was identified by manually contouring the endocortical region and then using a threshold of 300 mgHA/cm^3^ and 480 mgHA/cm^3^ to segment bone from soft tissue in the femur and tibia respectively. In the proximal tibia, trabecular bone architecture was analyzed in a 1000 µm (100 transverse slices) long region that began 100 µm inferior to the growth plate and extended distally. In the femur, trabecular bone architecture was analyzed in a region that began 200 µm superior to the distal femoral growth plate and extended proximally 1500 µm (150 transverse slices). The segmented images were analyzed with the Scanco trabecular bone morphology script to measure trabecular bone volume fraction (Tb. BV/TV, %), trabecular bone mineral density (Tb.BMD, mgHA/cm^3^), trabecular thickness (Tb.Th, mm), trabecular number (Tb.N, mm^-1^), trabecular separation (Tb.Sp, mm), and connectivity density (Conn.D, 1/mm³). Cortical bone morphology was analyzed in a 500 µm (50 transverse slices) region that began 2 mm superior to the distal tibiofibular junction and extended distally (for the tibia) or at the femoral mid-diaphysis (for the femur). Cortical bone was segmented using a threshold of 700 mgHA/cm^3^ and the standard Scanco script for cortical bone morphology was used to measure total cross-sectional area (Tt.Ar, mm^2^), cortical bone area (Ct.Ar, mm^2^), medullary area (Ma.Ar, mm^2^), bone area fraction (Ct.Ar/Tt.Ar, %), cortical tissue mineral density (Ct.TMD, mgHA/cm^3^), cortical thickness (Ct.Th, mm), cortical porosity (%), and the maximum, minimum, and polar moments of inertia (I_max_, I_min_, and J, mm^4^). The volume of the marrow cavity in four regions of the tibia was measured by subtracting the bone volume from the total volume (marrow volume = total volume – volume of bone).

**Mice:** At wean, (or for C57BL/6J mice upon arrival) all mice were group housed (n=2-5) with Alpha-Dri Plus Bedding (Shepherd Specialty Paper, TN, USA) in the SPF animal facility at MMCRI. Mice had ad libitum access to food (TEKLAD Global 2919 irradiated diet, ENVIGO, IN, USA) and autoclaved water in their home cages for the duration of the experiment. Naïve mice were randomly allocated to either group (vehicle or DT-treated) prior to DT injection on day 0. Treatment was administered by alternating vehicle and DT-treated cages. Group sizes were selected based on previous experiments within this model system. We observed 7 adverse events (sudden deaths) throughout the duration of these experiments; all in OCN-Cre;iDTR DT-treated animals (3 males and 4 females), and represent approximately 13.7% of DT-treated animals. These unexpected deaths of DT-treated animals occurred at various time points throughout the DT-dosing regimen (between days 2-12 of 14). No vehicle-treated OCN-Cre;iDTR mice, or C57BL/6J Vehicle- or DT-treated mice succumbed to sudden death during these experiments and, as such, we attribute these deaths to changes occurring as a result of OCN+ bone cells responding to DT treatment. In response to these events, we made three changes: (1) HydroGel and wet grain were added to the bottom of all experimental cages, (2) daily checks of DT-treated animals were executed and (3) metabolic cage experiments were carried out after the minimal amount of DT-treatments (1 week) to minimize exposure to detrimental effects and limit harm to animals.

**Supplemental Figure Legends**

**Supplemental Figures**

**Supplemental Figure 1. The effects of DT-treatment in OCN-Cre;iDTR female long bones.** Femoral histomorphometry confirmed reduced osteocyte numbers per total area (Ot.N/T.AR, #/mm^2^) (A), with no significant differences in osteoblast number (Ob.N/B.pm, #/mm) (B), or osteoclast number (Oc.N/B.pm, #/mm) (C) per bone perimeter, or in bone marrow adipose volume per total volume (AV/TV, %) (D). Trabecular bone volume per total volume (Tb. BV/TV, %) (E), trabecular number (Tb.N, 1/mm) (F) and trabecular spacing (Tb.Sp, mm) (G) were all significantly affected by DT-treatment in female mice. Cortical area (I), total cross-sectional area (J) and marrow area (K) as assessed by tibial µCT were unchanged with DT-treatment. Static histomorphometry data represents 8-10-week-old female mice, vehicle n=14, DT-treated n=9. Additional representative µCT images of tibial cross sections (H) from vehicle (top) and DT-Treated (bottom). **** p<0.001; *** p<0.001; ** p<0.01; * p<0.05 vs Vehicle. Data shown as individual dot plots ± S.D. All analyses were performed as a Student’s T-test within Prism.

**Supplemental Figure 2. The effects of DT-treatment in OCN-Cre;iDTR male long bones.** Femoral histomorphometry revealed no effect on osteocyte numbers per total area (Ot.N/T.AR, #/mm^2^) (A), and no significant differences in either osteoblast surface (Ob.S/BS, %) (B) or osteoclast surface (Oc.S/BS, %) (C) per bone surface, or osteoid surface per bone surface (OS/BS, %) (D). Static histomorphometry data represents 8-10-week-old male mice, vehicle n=8, DT-treated n=3. Additional representative µCT images of tibial cross sections (E, F) from vehicle (top) and DT-Treated (bottom). Cortical area (J), total cross-sectional area (K) and marrow area (L) as assessed by tibial µCT were unchanged with DT-treatment. Tibial µCT data represents 8-10-week-old male mice. Data shown as individual dot plots ± S.D.

**Supplemental Figure 3.** **Cartilage expansion in the presence of hypertrophic chondrocytes was characterized in the majority of OCN-Cre;iDTR male mice treated with DT.** Representative safranin O (red) stained image of DT-treated male (4x) showed extensive cartilage expansion into the femoral primary spongiosa (A). Zoomed in section (20x) along the growth plate highlighting hypertrophic chondrocytes (B), Scale bar = 100 μm. Four representative images (4x) of DT-treated males with increased cartilage expansion from the growth plate into the primary spongiosa with evidence of further femoral clubbing, as previously shown in Figure 3 (C). Scale bar = 0.5mm. Green arrows indicate abnormal region of hypertrophic chondrocytes and cartilage extending from the growth plate observed in males. Black arrows indicate a rounding or “clubbing” anatomic change in male femora. Serum RANKL (D) as measured by ELISA in vehicle (n=8) and DT-treated (n=6) male mice.

**Supplemental Figure 4. Chondrocyte expansion of the femur after DT-treatment proved to be sex-specific and non-mineralized cartilage.** Vehicle-treated females and males (left panel) showed normal cartilage staining (Safranin O, red) along the growth plate (A), while DT-treated males (lower right panel) showed an expansion of cartilage that was not observed in the DT-treated females (upper right panel) (A). Representative femoral histology images with von Kossa and Safranin O counterstain in DT-treated males showed cartilage expansion stemming from the growth plate was not mineralized, yet there was an increase in mineralized trabecular bone beyond the primary spongiosa (B). Images were taken at 4x magnification. Scale bar = 0.5 mm. White arrows indicate region of expansion of calcified matrix, only observed in male mice.

**Supplemental Figure 5. Body composition analysis of 8-week old female and male mice after 2 weeks of DT-treatment.** Weight change (%) in females (A) and males (B) throughout DT-treatment (n=3 females, n=3 males) compared to vehicle (n=3 females, n=3 males; PIXI analysis (n=3 per group per sex) of lean mass (g) in females (C) and males (D), fat mass (g) in females (E), and males (F). The ratio of fat mass/tissue mass (%) in females (G) and males (H). n=3. **** p<0.001; *** p<0.001; ** p<0.01; * p<0.05 vs Vehicle. Data are shown as individual dot plots ± S.D. All analyses were performed as a Student’s T-test within Prism.

**Supplemental Figure 6. DT-treatment in C57BL/6J mice shows no effect on metabolic activity.** Metabolic cage analysis was conducted following one-week of DT-treatment in 8-week old mice; n=5 per group. Average energy expenditure (EE) (kcal/hr) (A), resting energy expenditure (REE) (kcal/30 min) (B), active energy expenditure (AEE) (kcal/15 min) (C), respiratory quotient (RQ, %) (D), food consumption (g/24hr) (E), water consumption (g/day) (F), wheel distance (m/24h) (G), wheel speed (m/s) (H). 12 hour cycle data for time spent sleeping (hr) in the day (I) and night. **** p<0.001; *** p<0.001; ** p<0.01; * p<0.05 vs Vehicle. Data are shown as individual dot plots ± S.D. All analyses were performed as a Student’s T-test within Prism.

**Supplemental Figure 7. Decreased sclerostin levels could contribute to body weight reductions in DT-treated OCN-Cre;iDTR mice**. Osteocyte-secreted SOST is significantly decreased in circulation (blood serum) by day 7 of DT treatment as measured via rat/mouse ELISA (A); Vehicle (n=3), DT-treated (n=3), male mice aged 6 weeks at the start of treatments. Sclerostin immunohistochemistry effectively labeled osteocytes in vehicle treated animals (B) while the majority of osteocytes had been removed by 14 days of DT treatment (C), leaving empty canaliculi and very little SOST label (arrows); images of cortical bone from proximal femur taken at 40X; mice aged 6-8 weeks at the start of treatments.
